# Cellular Responses and Targets in Food Spoilage Yeasts Exposed to Antifungal Prenylated Isoflavonoids

**DOI:** 10.1128/spectrum.01327-23

**Published:** 2023-07-10

**Authors:** Sylvia Kalli, Cindy Vallieres, Joseph Violet, Jan-Willem Sanders, John Chapman, Jean-Paul Vincken, Simon V. Avery, Carla Araya-Cloutier

**Affiliations:** a Laboratory of Food Chemistry, Wageningen University & Research, Wageningen, the Netherlands; b School of Life Sciences, University of Nottingham, Nottingham, United Kingdom; c Unilever Foods Innovation Centre, Wageningen, the Netherlands; Agroscope

**Keywords:** antifungal, prenylated isoflavonoids, glabridin, wighteone, *Zygosaccharomyces parabailii*, *Saccharomyces cerevisiae*, chemogenomics, transcriptomics

## Abstract

Prenylated isoflavonoids are phytochemicals with promising antifungal properties. Recently, it was shown that glabridin and wighteone disrupted the plasma membrane (PM) of the food spoilage yeast Zygosaccharomyces parabailii in distinct ways, which led us to investigate further their modes of action (MoA). Transcriptomic profiling with *Z. parabailii* showed that genes encoding transmembrane ATPase transporters, including Yor1, and genes homologous to the pleiotropic drug resistance (PDR) subfamily in Saccharomyces cerevisiae were upregulated in response to both compounds. Gene functions involved in fatty acid and lipid metabolism, proteostasis, and DNA replication processes were overrepresented among genes upregulated by glabridin and/or wighteone. Chemogenomic analysis using the genome-wide deletant collection for S. cerevisiae further suggested an important role for PM lipids and PM proteins. Deletants of gene functions involved in biosynthesis of very-long-chain fatty acids (constituents of PM sphingolipids) and ergosterol were hypersensitive to both compounds. Using lipid biosynthesis inhibitors, we corroborated roles for sphingolipids and ergosterol in prenylated isoflavonoid action. The PM ABC transporter Yor1 and Lem3-dependent flippases conferred sensitivity and resistance, respectively, to the compounds, suggesting an important role for PM phospholipid asymmetry in their MoAs. Impaired tryptophan availability, likely linked to perturbation of the PM tryptophan permease Tat2, was evident in response to glabridin. Finally, substantial evidence highlighted a role of the endoplasmic reticulum (ER) in cellular responses to wighteone, including gene functions associated with ER membrane stress or with phospholipid biosynthesis, the primary lipid of the ER membrane.

**IMPORTANCE** Preservatives, such as sorbic acid and benzoic acid, inhibit the growth of undesirable yeast and molds in foods. Unfortunately, preservative tolerance and resistance in food spoilage yeast, such as Zygosaccharomyces parabailii, is a growing challenge in the food industry, which can compromise food safety and increase food waste. Prenylated isoflavonoids are the main defense phytochemicals in the Fabaceae family. Glabridin and wighteone belong to this group of compounds and have shown potent antifungal activity against food spoilage yeasts. The present study demonstrated the mode of action of these compounds against food spoilage yeasts by using advanced molecular tools. Overall, the cellular actions of these two prenylated isoflavonoids share similarities (at the level of the plasma membrane) but also differences. Tryptophan import was specifically affected by glabridin, whereas endoplasmic reticulum membrane stress was specifically induced by wighteone. Understanding the mode of action of these novel antifungal agents is essential for their application in food preservation.

## INTRODUCTION

**FIG 1:**
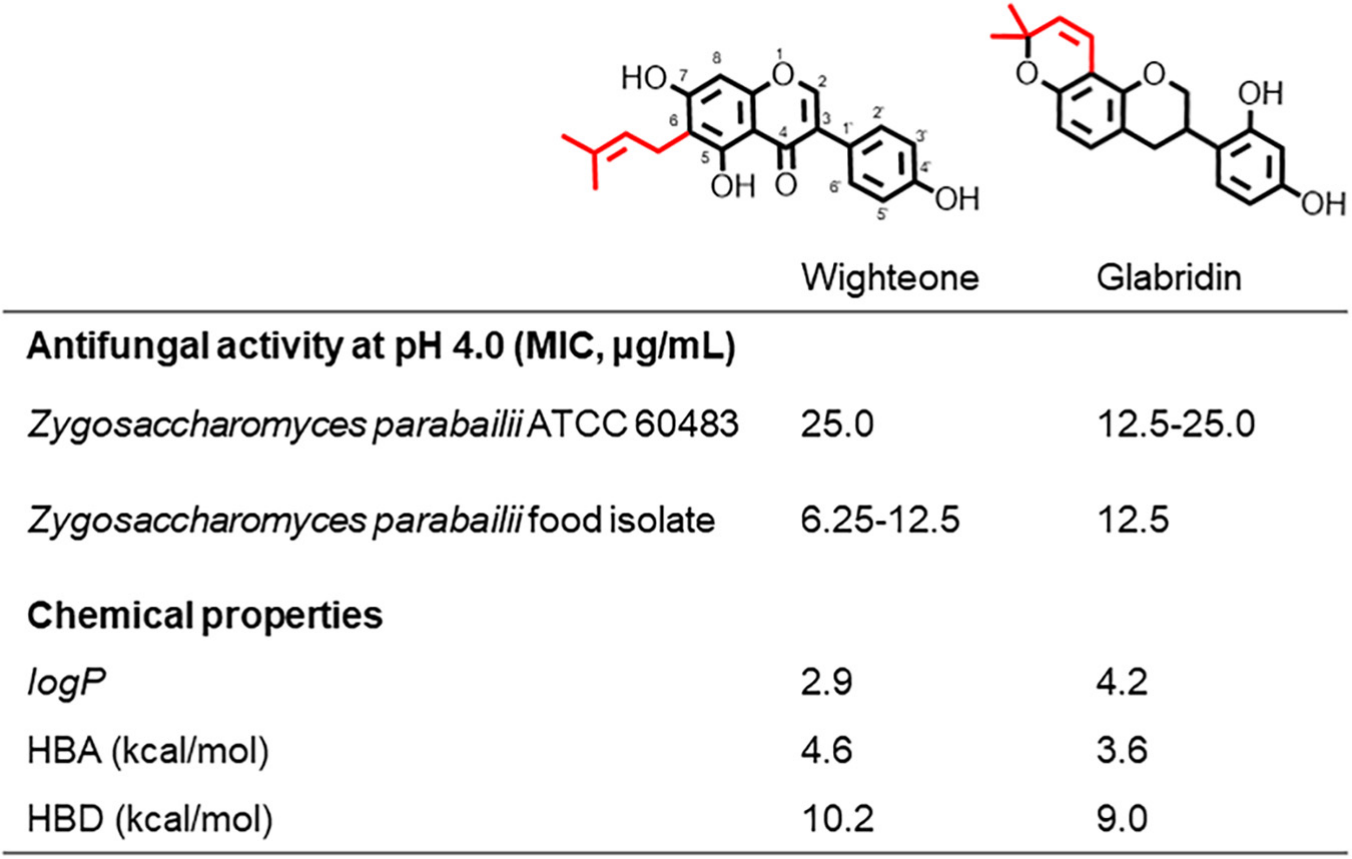
Molecular structure, antifungal activity against strains of *Z. parabailii* at pH 4.0 (MIC values from reference 11) and relevant physicochemical properties, namely, hydrophobicity (logP) and hydrogen bond (HB) acceptor (A) and donor (D) capacity, of the prenylated isoflavonoids wighteone and glabridin. The IUPAC numbering is the same for the two prenylated isoflavonoids.

The quest for novel, natural food preservatives has spurred a focus on plant metabolites with antimicrobial properties. Prenylated flavonoids and isoflavonoids [i.e., (iso)flavonoids] are biosynthesized in legumes as part of their defense mechanism against pathogens. Their antimicrobial properties have already been documented *in vitro* against Gram-positive and Gram-negative bacteria (1–4), molds (5, 6) and yeasts (7–10). (Iso)flavonoids are a chemically diverse group of phenyl benzopyrans encompassing 21 different subclasses, mainly differing in the level of oxidation of the middle ring. The chemical diversity of (iso)flavonoids is further increased through the different type, number and configuration of substituents that decorate the backbone. Prenyl groups (i.e., C_5_-isoprenoid moieties) are a typical decoration of (iso)flavonoids and can adopt a chain or ring configuration. In recent work, we showed that two prenylated isoflavonoids, the chain-prenylated isoflavone wighteone and the ring-prenylated isoflavan glabridin (Fig. 1) were highly effective in inhibiting the notorious food spoilage yeast Zygosaccharomyces parabailii (11). Their MICs (between 6.3 and 25.0 μg/mL) were over 10-fold lower than that of the traditional preservative of acidic food products, sorbic acid (11). In the same work, we also demonstrated that these two compounds disrupted the plasma membrane (PM) integrity of the yeast, yet with different phenotypes. Upon wighteone exposure, membrane permeabilization (measured by propidium iodide uptake) coincided with killing, whereas upon glabridin exposure, killing preceded propidium iodide uptake (11). Furthermore, *Z. parabailii* cells treated with equitoxic amounts of wighteone showed endocytosis-like deformations and membrane discontinuities upon short (15 min) exposure and complete disappearance of the membrane after long exposure (180 min), whereas cells treated with glabridin showed PM discontinuities that intensified over time (up to 180 min) (11).

Prenylation is generally accepted to confer hydrophobicity to the molecules, increasing their affinity to biological targets (including the PM). However, not all prenylated compounds are active antimicrobials and their hydrophobicity index (logP) is not always proportional to their activity. Prior work highlighted that active prenylated (iso)flavonoids with different molecular properties can potentially have different interactions with the membrane or even mode of action (MoA) (12). Glabridin and wighteone have distinct predominant chemical properties. In particular, glabridin has 20-fold-higher hydrophobicity and more than 10-fold-lower hydrogen bonding (HB) capacity than wighteone (12) (Fig. 1). Interestingly, Araya-Cloutier et al. (4) showed that wighteone is a good permeabilizer of the Gram-positive bacterium Listeria monocytogenes, whereas glabridin or other active mono- and diprenylated (iso)flavonoids were poor permeabilizers. Other studies have reported cytosolic presence and activity of diprenylated (iso)flavonoids (13, 14). das Chagas Almeida et al. (15) demonstrated that the antimicrobial activity of a plant extract rich in mono- and diprenylated (iso)flav(an)ones was exerted without permeabilization of the PM of Staphylococcus aureus (assessed through crystal violet uptake at extract concentrations up to 8× MIC) or any disruption of the cell surface (as evidenced by atomic force microscopy at the MIC), suggesting alternative targets in bacteria.

Few studies have used genetic or genomic tools to gain insights into the MoA of prenylated (iso)flavonoids. Moazeni et al. (16) selectively investigated the effect of glabridin on inducing apoptosis by studying two apoptosis-related genes in Candida albicans, *MAC1* and *NUC1*. The authors reported elevated expression of these two genes in response to glabridin. Yin et al. (17) performed a more complete transcriptome profiling of wighteone-treated S. cerevisiae cells. Genes regulating peptide transport systems through the pleiotropic drug resistance (PDR) network and endoplasmic reticulum (ER) stress were activated in response to wighteone. The aim of Yin’s study was to differentiate wighteone’s MoA from those of commonly used antifungal drugs, but mechanistic details on wighteone’s MoA were limited. In the present study, we aimed to study in more detail the mode of antifungal action of glabridin and wighteone and potentially to highlight any differences. For this, we employed transcriptome profiling of the notorious food spoilage yeast *Z. parabailii* (whose genome sequence has recently been fully described [18]) exposed to the two active prenylated isoflavonoids, complemented by a genome-wide chemogenomic analysis using the full deletion strain collection available for the model yeast Saccharomyces cerevisiae (19).

## RESULTS

To identify possible leads to the MoA of the active antifungal prenylated isoflavonoids, wighteone and glabridin, transcriptomic analysis and gene deletant (chemogenomic) screening were carried out separately against these compounds. Key genes identified from these analyses were corroborated in independent phenotypic tests.

### Transcriptomic profiling of *Z. parabailii* cells treated with prenylated isoflavonoids.

Transcriptome profiling was performed for exponential-phase *Z. parabailii* cells after 30- and 120-min exposures to doses of glabridin or wighteone that gave a mild yeast inhibition effect (>85% of survival compared to the control after 120 min). In total, around 1,450 and 2,280 genes were significantly (*P* < 0.05) differentially expressed in response to wighteone and glabridin, respectively, at both time points tested (see volcano plots in Fig. S2 and Table S1C in the supplemental material). Wighteone exposure was associated with upregulation (according to log_2_ fold change of >1.0) of 62 genes (i.e., 4% of the significant differentially expressed genes) and 169 (12%) genes after 30 and 120 min of exposure, respectively. Glabridin significantly upregulated 125 (5%) and 418 (18%) genes after the short and the long exposures, respectively. Wighteone exposure was associated with downregulation (log_2_ fold change of less than −1.0) of 51 (4%) and 66 (5%) genes after 30 and 120 min of exposure, respectively, whereas glabridin significantly downregulated 58 (3%) and 104 (5%) genes.

**FIG 2:**
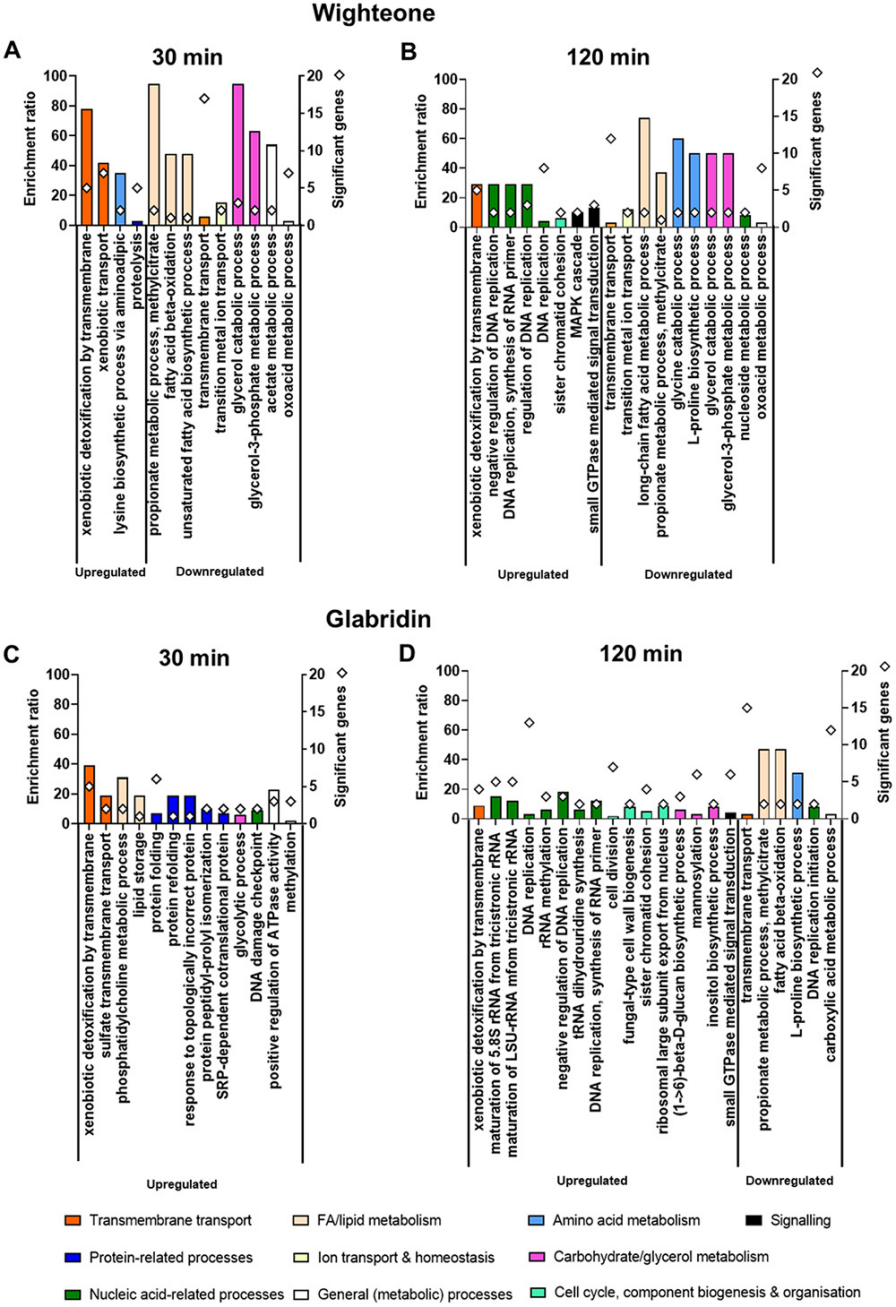
Up- and downregulated GO terms (biological processes) in *Z. parabailii* (ATCC 60483) cells upon exposure to wighteone (A and B) and glabridin (C and D) after 30 min (A and C) and 120 min (B and D), respectively. GO terms encompass genes with both a significant (*P* < 0.05) and log_2_ fold change of >|1.0|. Biological process categories broader than those shown on the figure’s *x* axes are differentiated by color, as defined by the legend below the panels. Only nonredundant GO terms are shown (a semantic similarity threshold of <0.7 was used according to reference 80). Diamonds represent the number of significantly up- or downregulated genes per overrepresented biological function. Genes represented by the different GO terms, together with their description and significance (*P* value), are shown in Table S2.

Figure 2 illustrates overrepresented Gene Ontology (GO) terms in the annotations of genes found in the up- or downregulated gene sets by the two prenylated isoflavonoids at the different time points of exposure, as identified by the GO analysis. In general, the enrichment ratios for biological processes affected by wighteone were higher than those affected by glabridin at similar exposure times. The GO analysis highlighted that ATPase transmembrane transport, including xenobiotic detoxification, were uniformly overrepresented GO terms (biological processes) in the annotations of genes upregulated by both compounds and time points. Genes homologous to the S. cerevisiae multidrug ATPase transporter subfamily Pdr5/Pdr15/Pdr10 (20) and the ATPase transmembrane transporter Yor1 involved in antibiotic oligomycin resistance (21) are known representatives of this GO term (Table S2). Other GO terms for upregulated genes differed between wighteone and glabridin. The gene set upregulated after 30 min of exposure to wighteone was enriched with lysine biosynthesis and proteolysis functions (Fig. 2A) and, after 120 min of exposure, with functions related to DNA replication among others (Fig. 2B). Genes upregulated by glabridin after 30 min were overrepresented by functions associated with transmembrane sulfate transport, phosphatidylcholine (PC) biosynthesis, and proteostatic functions, such as protein (re)folding, protein peptidyl-prolyl isomerization, and response to topologically incorrect protein (Fig. 2C). After 120 min of exposure to glabridin, nucleic acid-related processes and cell cycle, component biogenesis, and organization were among the overrepresented functional categories in the upregulated gene set (Fig. 2D).

The GO analysis also revealed several biological processes overrepresented in the annotations of genes downregulated by the two prenylated isoflavonoids. After 30 min of exposure to wighteone, we observed downregulation of glycerol catabolism and of fatty acid (FA) or lipid metabolism, including desaturation (Fig. 2A) (through downregulation of the sole Δ^9^ FA desaturase of S. cerevisiae, Ole1) (Table S2). Transmembrane transport processes were also overrepresented after 30 min. Similar processes were overrepresented in the downregulated gene set after 120 min of wighteone exposure, along with amino acid (glycine and proline) metabolism (Fig. 2B). Interestingly, no overrepresented gene functions were downregulated after 30 min of exposure to glabridin (Fig. 2C), but functions associated with propionate and FA metabolism and proline biosynthesis were downregulated after 120 min. More than 15 genes associated with transmembrane transport were downregulated after 120 min of glabridin exposure, although with a low enrichment ratio (Fig. 2D).

Overall, several biological processes appeared to be affected (up- or downregulated) by the two active prenylated isoflavonoids, the most dominant being (i) (xenobiotic) transmembrane transport, (ii) fatty acid and lipid metabolism, and (iii) proteostatic processes in the 30-min exposure, succeeded by nucleic acid-related processes in the 120-min exposure. Wighteone additionally affected (downregulated) processes related to glycerol metabolism.

### Yeast deletion strains sensitive and resistant to the prenylated isoflavonoids.

**FIG 3:**
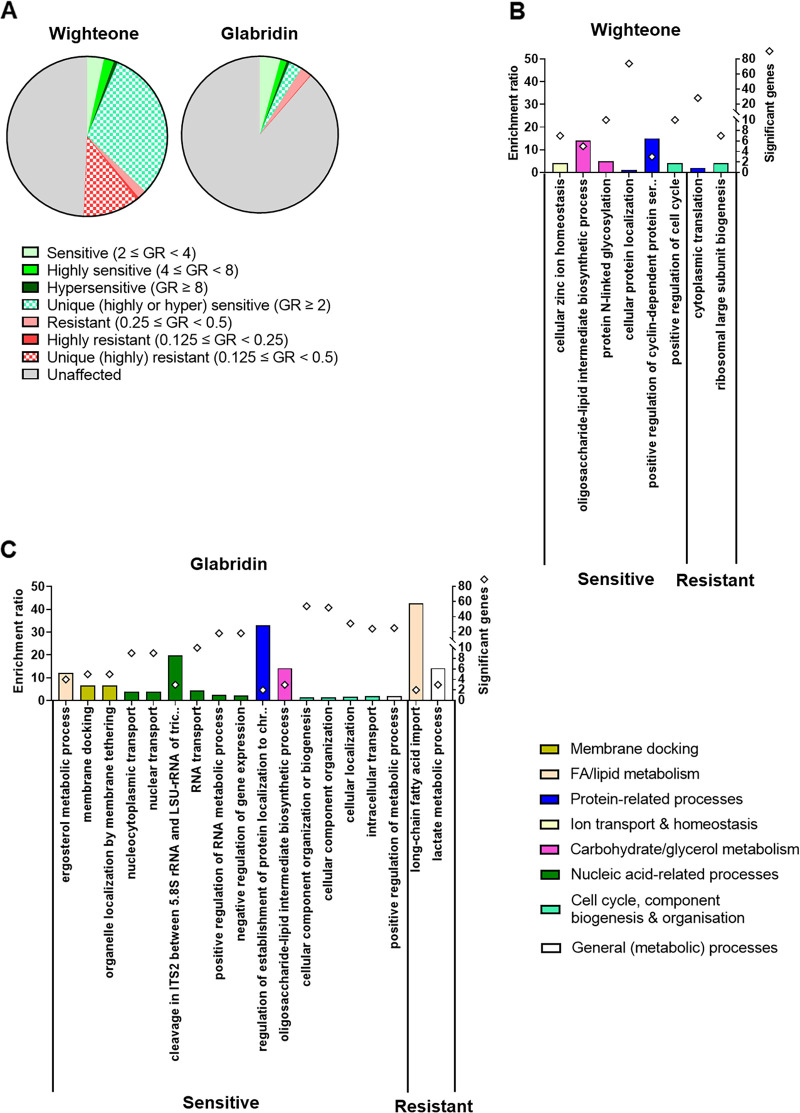
Overview of chemogenomic screening using S. cerevisiae deletants. (A) Distribution of sensitive and resistant deletants to both prenylated isoflavonoids (solid green and red, respectively, with darker colors denoting a higher level of sensitivity or resistance) together with the fractions of affected deletants that were unique for each prenylated isoflavonoid (patterned colors). Unaffected deletants are depicted in gray. (B and C) GO enrichment analysis for biological processes in the annotations of significant genes (*P* < 0.05) where deletion produced sensitive (GR > 2.0) or resistant (GR ≤ 0.5) phenotypes to wighteone and glabridin. Broader biological process categories than those shown on the figures’ *x* axes are differentiated by color, as defined by the legend at the bottom right of the figure. Only nonredundant GO terms are shown (a semantic similarity threshold of <0.7 was used according to reference 80). Diamonds represent the number of significant genes per overrepresented biological function. Genes represented by the different GO terms together with their description and significance (*P* value) are shown in Table S3.

To complement the transcriptomic profiling, a chemogenomic analysis was performed. This aspect used another spoilage (and model) yeast, S. cerevisiae, capitalizing on the genome-wide collection of homozygous deletant strains available for this organism. Figure 3 depicts an overview of the results. In total, around 2,000 and 500 deletion strains showed altered growth phenotypes (either sensitivity at a growth rate [GR] of >2.0 or resistance at a GR of ≤0.5) when cultured for 24 h with wighteone and glabridin, respectively (Fig. 3A). The sensitive phenotypes were more common than the resistant phenotypes, irrespective of the prenylated isoflavonoid. There were 273 deletants commonly sensitive and 100 deletants commonly resistant to both prenylated isoflavonoids (Fig. 3A). A GO analysis pinpointed several biological processes that were overrepresented among the annotations of genes whose deletions produced altered phenotypes (Fig. 3B and C). The most highly enriched biological processes were associated with genes whose deletion yielded sensitivity (i.e., genes required for wild-type [WT] resistance) to wighteone were oligosaccharide-lipid intermediate biosynthesis related to mannoprotein synthesis and activation of cyclin-dependent protein kinase activity (Fig. 3B). Deletants for 74 genes associated with cellular protein localization processes gave sensitive phenotypes to wighteone, albeit with a low enrichment ratio (1.4) (Fig. 3B). In contrast, deletants of genes involved in cytoplasmic translation (i.e., protein biosynthesis) and ribosome biogenesis were overrepresented among deletants giving resistance phenotypes (i.e., genes conferring sensitivity) to wighteone (Fig. 3B). In the case of glabridin, deletants of functions associated with regulation of protein localization to chromosome were overrepresented among deletants giving sensitive phenotypes, albeit based on only two significant genes. Moreover, deletants of genes involved in RNA-related processing, oligosaccharide-lipid intermediate biosynthesis, and ergosterol biosynthesis as well as membrane docking were also highly enriched, representing significant terms among the sensitive GO terms. Fatty acid import was the most significantly enriched biological process associated with genes whose deletion yields resistance to glabridin, although this was based on only two significant genes (Fig. 3C).

### Roles of fatty acid import and lipid biosynthesis functions in responses to prenylated isoflavonoids.

**FIG 4:**
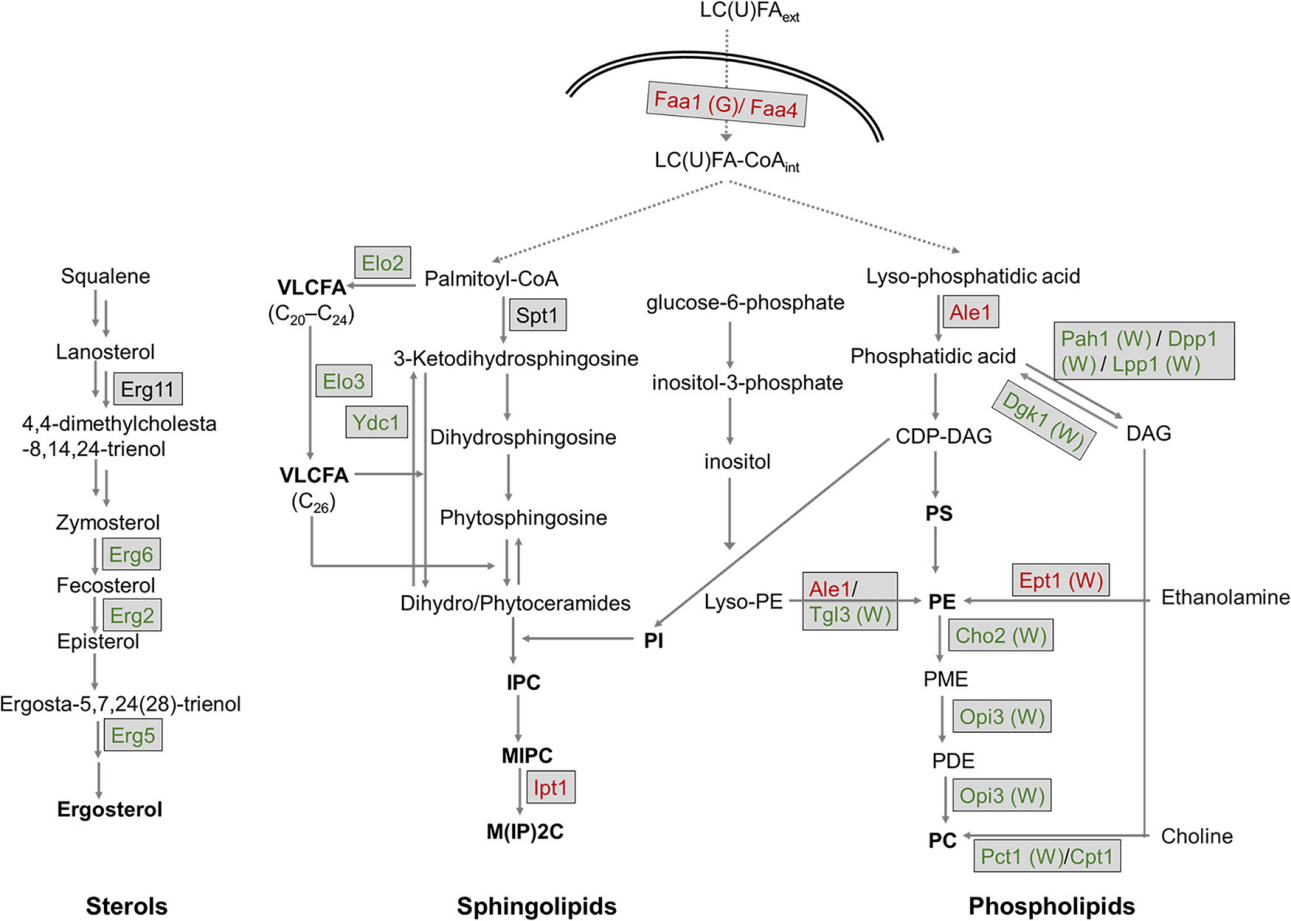
Intracellular biosynthesis pathways for ergosterol, sphingolipids, and phospholipids in S. cerevisiae based on references 84 to 86. In the case of blocked sphingolipid or phospholipid biosynthesis, long-chain (unsaturated) fatty acids [LC(U)FA] are imported to the cell (dashed arrows). Proteins involved in the biosynthesis of the three major lipid classes are colored based on the observed phenotype of their gene deletants upon exposure to the two prenylated isoflavonoids according to the chemogenomic screening: green indicates sensitive deletants, whereas red shows resistant deletants. “(W)” or “(G)” indicates that the specific deletant phenotype was only observed in response to either wighteone or to glabridin, respectively. The corresponding GR and *P* values of the deletants can be found in Table S4. Faa1 and Faa4, long-chain fatty acyl-CoA synthetases; Erg2, Δ^8^-sterol isomerase; Erg5, Δ^22^-sterol desaturase; Erg6, Δ^24^-sterol C-methyltransferase; Erg11, lanosterol 14α-demethylase; Spt1, serine C-palmitoyltransferase; Ydc1, alkaline dihydroceramidase; Ipt1, inositol phosphotransferase; Elo2, FA elongase of sphingolipid biosynthesis, which acts on FAs of up to C_24_ FAs from C_18_-CoA; Elo3, FA elongase of sphingolipid biosynthesis, which acts on FAs of up to C_20_ to C_26_ FAs from C_18_-CoA; Ale1, lysophospholipid acyltransferase; Pah1, phosphatidic acid (PA) phosphatase; Dpp1, diacylglycerol pyrophosphate; Lpp1, lipid phosphate phosphatase; Tgl3, bifunctional triacylglycerol lipase and lysophosphatidylethanolamine acyltransferase; Ept1, *sn*-1,2-diacylglycerol ethanolamine and choline phosphotransferase; Cho2, phosphatidylethanolamine methyltransferase; Opi3, methylene-fatty acyl-phospholipid synthase; Pct1, choline phosphate cytidylyltransferase; Cpt1, choline phosphotransferase; VLCFA, very-long-chain fatty acids; IPC, inositol phosphoryl ceramide; MIPC, mannosyl inositol phosphoryl ceramide; M(IP)2C, mannosyl-di-(inositolphosphoryl)ceramide; PI, phosphoinositol; (CDP)-DAG, (CDP) diacylglycerol; PS, phosphatidylserine; P(M)E, (*N*-methyl)phosphatidylethanolamine; PDE, phosphatidyldimethylethanolamine; PC, phosphatidylcholine.

Due to the hydrophobic nature of prenylated isoflavonoids, it was anticipated that deletion of genes important in lipid biosynthesis may yield altered phenotypes. In line with this, the GO analysis showed that ergosterol biosynthesis and long-chain fatty acid (LCFA) intracellular import were significant biological processes that, when perturbed, tended to yield altered phenotypes in response to glabridin (Fig. 3B). Figure 4 shows the biosynthetic pathways of the three main lipid classes, highlighting in color the genes that gave altered phenotypes with the prenylated isoflavonoids (resistance in red and sensitivity in green). Deletants lacking genes involved in the late steps of ergosterol biosynthesis, *ERG2*, *ERG5*, and *ERG6* (being the only viable *erg* deletants) (22) were all highly sensitive (GR > 6.7; *P* ≤ 1E−04) to both glabridin and wighteone (Table S4). In contrast, deletants lacking one or both of the fatty acyl coenzyme A (acyl-CoA) synthetases encoded by *FAA1* and *FAA4*, which import LCFAs, were resistant to prenylated isoflavonoids (GR*_faa1_*_Δ_ = 0.5 and *P* = 5E−03 for glabridin but insignificant for wighteone; GR*_faa4_*_Δ_ = 0.3 and 0.5 for wighteone and glabridin, respectively; *P* ≤ 1E−03) (Table S4). These two synthetases import (un)saturated FAs (23) (up to C_20_) (24) into the cell from the extracellular space and subsequently activate them, when the intracellular synthesis of the sphingo- and phospholipid precursors is blocked (22).

Along with ergosterol biosynthesis and import/activation of LCFAs, altered phenotypes of deletants of sphingolipid and phospholipid biosynthesis were also observed. The deletion strain for *IPT1*, which encodes the inositolphosphotransferase catalyzing the final biosynthetic step of the most abundant, terminal sphingolipid, mannosyl-di-(inositolphosphoryl)-ceramide [M(IP)2C] from mannosylinositol phosphorylceramide (MIPC) (Fig. 4), was resistant to both compounds (GR = 0.2 for wighteone and GR = 0.5 for glabridin; *P* ≤ 4E−03) (Table S4). Consistent with this observation, the *ydc1*Δ strain, lacking the ceramidase that hydrolyzes the dihydroceramide to dihydrosphingosine (thus going backwards to sphingolipid precursors) (Fig. 4) (22) was hypersensitive to both compounds (GR = 29.8 for wighteone and GR = 39.0 for glabridin; *P* ≤ 7E−07) (Table S4). Furthermore, deletants of the FA elongases that extend C_16_-to-C_18_ FAs to up to C_20_ to C_26_ (25), *ELO2* and *ELO3*, were also sensitive to both compounds in the chemogenomic screening (GR*_elo2_*_Δ_ = 3.9 to 5.3 and GR*_elo3_*_Δ_ 2.5 to 3.6; *P* ≤ 3E−03) (Table S4).

Several deletants of phospholipid biosynthetic genes (Fig. 4) showed altered phenotypes, particularly in response to wighteone. For example, the phospholipid biosynthesis-related *dgk1*Δ deletant lacking a diacylglycerol (DAG) kinase was hypersensitive to wighteone, but not to glabridin (GR = 28.0; *P* 3E−06) (Table S4). Moreover, the *cpt1*Δ deletant, lacking a gene important for PC biosynthesis, and the *tgl3*Δ deletant, defective for phosphatidylethanolamine (PE) biosynthesis, were either more sensitive to wighteone than to glabridin (GR*_cpt1_*_Δ_ = 5.1 and *P* = 1E−03 for wighteone and GR*_cpt1_*_Δ_ = 2.7 and *P* = 7E−03 for glabridin) (Table S4) or highly sensitive only to the former (GR*_tgl3_*_Δ_ = 4.7 and *P* = 6E−06 for wighteone) (Table S4). Similarly, *ino2*Δ and *ino4*Δ strains lacking the transcription factors necessary for the derepression of PC biosynthetic enzymes in response to inositol depletion, Opi3 and Cho2, were also specifically sensitive to wighteone (GR = 4.4 and 4.9, respectively; *P* ≤ 1E−04) (Table S4).

**FIG 5:**
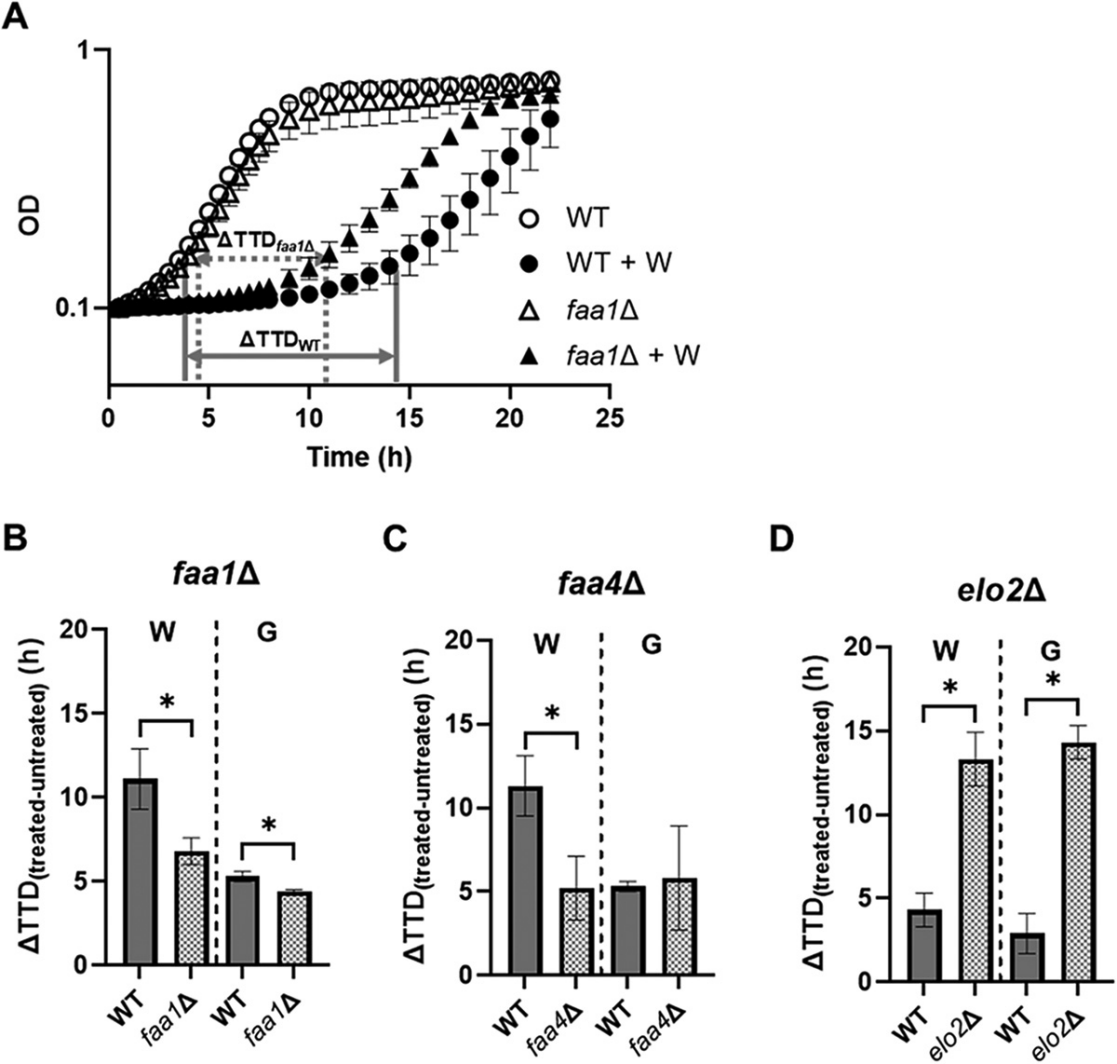
Resistance of S. cerevisiae deletants lacking the genes encoding the fatty acyl-CoA synthetases FAA1 (A and B) and FAA4 (C) and sensitivity of deletants lacking the gene encoding the FA elongase, *ELO2* (D), to the prenylated isoflavonoids wighteone (W) and glabridin (G). Treated S. cerevisiae *faa1*Δ and *faa4*Δ strains were cultured in the presence of 5.0 μg/mL wighteone or 10.0 μg/mL glabridin, whereas the treated *elo2*Δ strain was cultured in the presence of 3.8 μg/mL wighteone or 7.5 μg/mL glabridin. Filled bars represent the WT strain, and patterned bars represent the deletant strains. Values are the means ± standard deviation (SD) from three biological replicates, each performed in triplicate. Asterisks denote significant differences (*P* < 0.05). For the full growth curves, refer to Fig. S3A to D.

To corroborate the importance of long-chain fatty acid import in the yeast response to wighteone and glabridin, *faa1*Δ, *faa4*Δ, and *elo2*Δ deletants were tested in dedicated assays. Figure 5A shows growth curves for the *faa1*Δ deletant with or without wighteone (refer to Fig. S3 for all growth curves). With wighteone, *faa1*Δ cells showed faster growth than the wild type (WT). As much of the effect seemed to be a faster transition from lag to exponential growth, the phenotype can also be captured with the change in time to detection (ΔTTD) with and without an antimicrobial. For wighteone, the ΔTTD*_faa1_*_Δ_ and ΔTTD*_faa4_*_Δ_ were significantly smaller (*P* < 0.05) (Table S5) than that of ΔTTD_WT_, whereas for glabridin, only the ΔTTD*_faa1_*_Δ_ was significantly smaller (*P* < 0.05) (Table S5) than that of ΔTTD_WT_ (Fig. 5B and C). Hence the *faa1*Δ strain was resistant to both prenylated isoflavonoids and especially to wighteone (Fig. 5B; Fig. S3A compared to B), whereas the *faa4*Δ strain was resistant only to wighteone under this assay condition. In the earlier screen, the *elo2*Δ deletant gave the strongest phenotypes among deletants lacking a gene involved in FA elongation. This deletant was confirmed to be significantly hypersensitive (*P* < 0.05) (Table S5) to both prenylated isoflavonoids in the dedicated growth assays (Fig. 5D).

**FIG 6:**
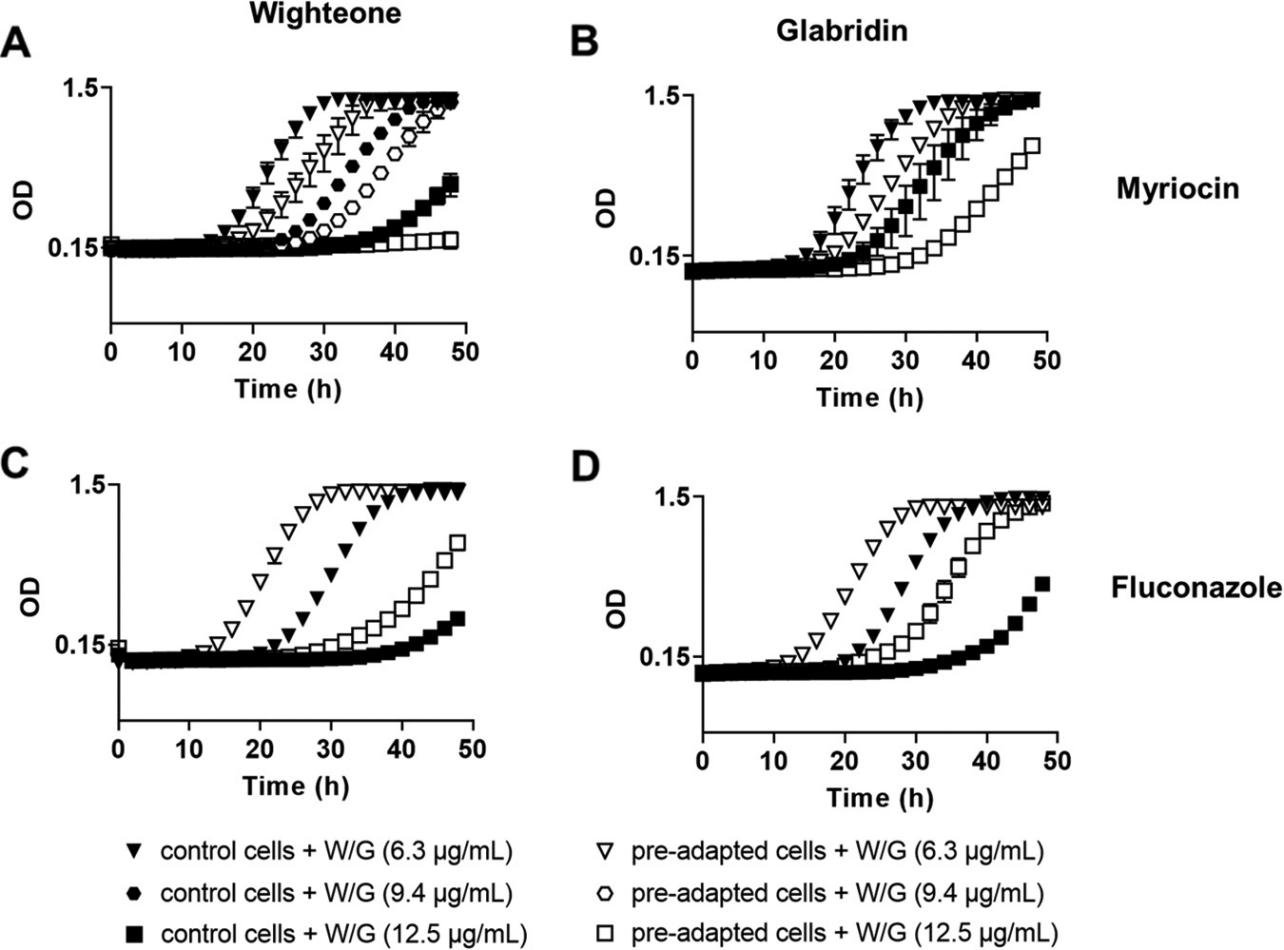
Effect of wighteone (W) and glabridin (G) on the growth of *Z. parabailii* (ATCC 60483) cells preadapted to myriocin (A and B) and fluconazole (C and D). Different symbols indicate different prenylated compound concentrations. The depicted curves are an example of the three biological replicates (data points are the means ± SD from technical duplicates). All biological replicates can be found in Fig. S4 and S5. Control cells and cells preadapted to myriocin or fluconazole without the addition of wighteone or glabridin are shown in Fig. S1C and D, respectively.

To corroborate that sphingolipid and ergosterol biosynthesis may affect the toxicity of glabridin and wighteone against *Z. parabailii* cells, cells were grown in the presence of subinhibitory concentrations of biosynthetic inhibitors (which did not affect the normal growth of the cells) (Fig. S1C and D) and prenylated isoflavonoids. We used the sphingolipid biosynthesis inhibitor myriocin, which inhibits the serine C-palmitoyltransferase (Spt1), the first enzyme in sphingolipid biosynthesis (26) (Fig. 4), and the ergosterol biosynthesis inhibitor fluconazole, which inhibits the lanosterol C_14_-demethylase Erg11, leading to accumulation of methylated early intermediates (27). Myriocin-preadapted yeast cells were more sensitive to the prenylated compounds than the control cells (Fig. 6A and B), supporting a role of sphingolipids in resistance to the prenylated isoflavonoids. In contrast, fluconazole-preadapted cells were more resistant to both compounds than the control cells (Fig. 6C and D). This suggests that blocking ergosterol biosynthesis at an early stage limited the toxicity of the prenylated isoflavonoids, contrasting with the sensitivity observed in deletants defective for later ergosterol biosynthesis steps.

Overall, the results indicated that phospholipid biosynthesis predominantly influences wighteone toxicity, whereas sphingolipid and ergosterol biosynthesis can influence the toxicity of both wighteone and glabridin.

### Role of transmembrane transport proteins in response to prenylated isoflavonoids.

From the chemogenomic data, intact biosynthesis of PM lipid components appeared to be important for S. cerevisiae to resist toxicity of prenylated isoflavonoids, corroborating the long-standing notion of PM activity of prenylated isoflavonoids (28–31). As the yeast PM is a host environment for protein transport systems, we considered data for deletants lacking PM transport systems such as the ATPase binding (ABC) transporters typically involved in xenobiotic detoxification (upregulated in our transcriptomic analysis), as reviewed by Paumi et al. (32). From the screen, all seven deletants of ABC plasma membrane transporters were sensitive to wighteone (Table S6). In particular, the *yor1*Δ and *pdr5*Δ strains yielded the most sensitive phenotypes in response to either one or both prenylated isoflavonoids among the deletants of known ABC plasma membrane transporters (GR*_yor1_*_Δ_ > 8.0 and *P* < 4E−05 in response to both compounds and GR*_pdr5_*_Δ_ = 18.9 and *P* = 2E−05 only in response to glabridin).

The Yor1 and Pdr5 proteins are involved in xenobiotic detoxification but are also known as floppases, which move phospholipids from the inner to the outer leaflet of the PM (21). The action of floppases is balanced by the action of flippases, such as P4-ATPases, the dimeric Lem3-Dn1f, or Lem3-Dn2f, which catalyze the inward movement of phospholipids, from the outer to the inner leaflet of the PM. Dnf1 and Dnf2 have redundant functions in yeast (33), and the phenotypes of their single deletants are typically phenocopied by the *lem3*Δ deletant (34, 35). In this study, the *lem3*Δ deletant showed GRs of 11.4 (*P* = 6E−06) and 3.2 (*P* = 3E−04) in the chemogenomic screening (Table S3), indicating a strong sensitization to wighteone and glabridin, respectively.

**FIG 7:**
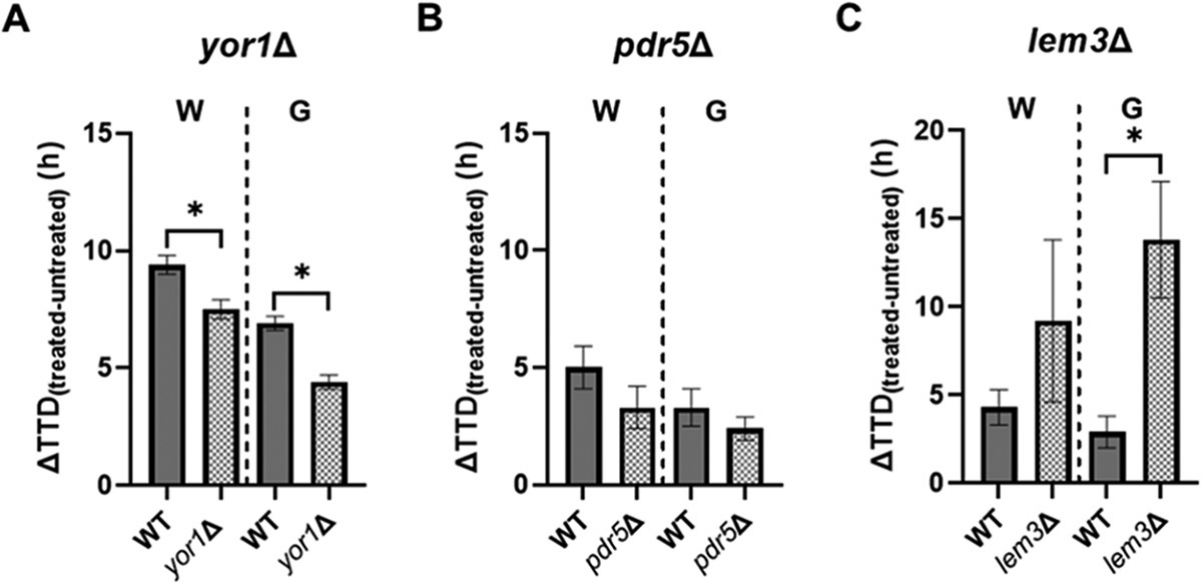
Phenotypes of ATPase transmembrane transporter *yor1*Δ, *pdr53*Δ, and *lem3*Δ deletion strains, to wighteone (W) or glabridin (G). Treated S. cerevisiae *yor1*Δ (A) and *pdr5*Δ (B) strains were cultured in the presence of 3.8 μg/mL wighteone or 7.5 μg/mL glabridin and the *lem3*Δ strain (C) in the presence of 5.0 μg/mL wighteone or 10.0 μg/mL glabridin. Filled bars represent the WT strain, and patterned bars represent the deletant strains. Values are the means ± SD from three biological replicates, each performed in triplicate. Asterisks denote significant differences (*P* < 0.05). For the full growth curves, refer to Fig. S6.

Figure 7 shows the growth phenotypes of ATPase transmembrane transporter *yor1*Δ, *pdr5*Δ, and *lem3*Δ deletants. In these dedicated growth assays with continuous shaking, the *yor1*Δ strain was found to be significantly (*P* < 0.05) (Table S5) resistant to both compounds (Fig. 7A; corresponding full growth curves in Fig. S6A and B), whereas the *pdr5*Δ strain did not yield strong phenotypes to either of the two prenylated isoflavonoids (Fig. 7B; Fig. S6C and D). This contrast with the screen results suggests condition dependency of these phenotypes (e.g., level of aeration). On the other hand, the growth of the *lem3*Δ deletant was significantly (*P* < 0.05) (Table S5) sensitive to glabridin, although a sensitive trend was observed for wighteone (Fig. 7C; Fig. S6). Together these results indicate that the ATPase transmembrane transporters Yor1, Pdr5, and Lem3 can influence the resistance to the prenylated isoflavonoids (with the *yor1*Δ and *pdr5*Δ phenotypes possibly being condition dependent).

**FIG 8:**
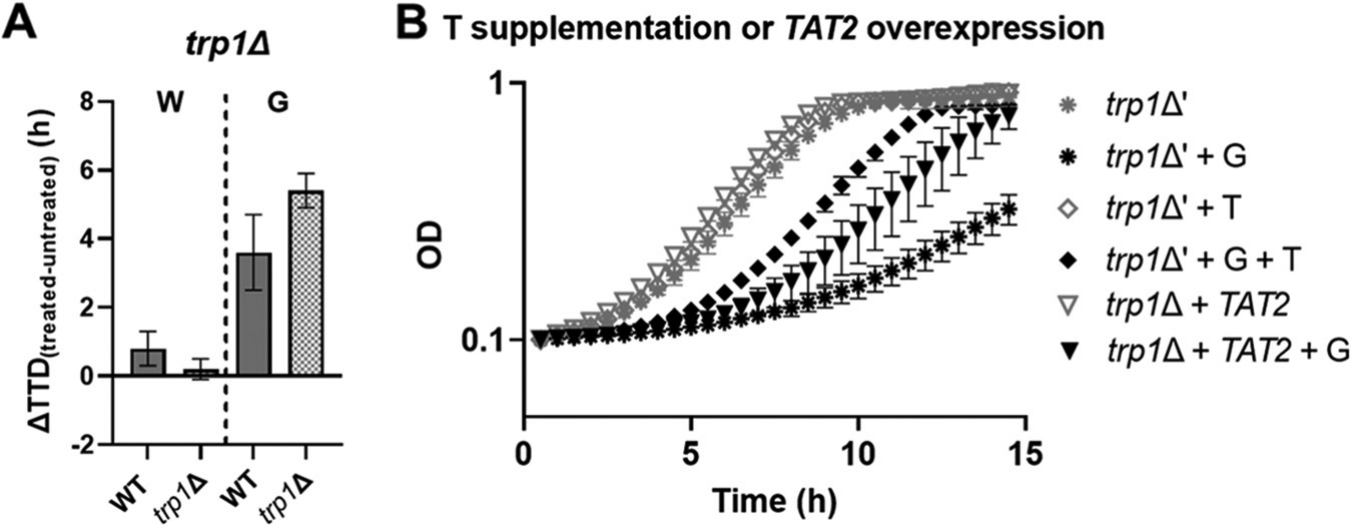
Sensitivity of *trp1*Δ cells to glabridin (G [7.5 μg/mL]), but not to wighteone (W [3.8 μg/mL]), (A) and rescue by *TAT2* overexpression or tryptophan (T) supplementation (B). Treated S. cerevisiae WT, *trp1*Δ and *trp1*Δ′ (*trp1*Δ plus empty vector) strains were cultured in the presence of 1 mM tryptophan and/or 7.5 μg/mL glabridin. The values shown are the means ± SD from three biological replicates, each performed in triplicate. For the full growth curves, refer to Fig. S7A and B.

The action of flippases/floppases and membrane asymmetry have an influence in many biological functions. Previously, it was shown that Lem3 and Yor1 impact the function of Tat2, the main PM transporter of tryptophan at tryptophan concentrations normally found in YPD (yeast extract-peptone-dextrose) medium (36, 37). We therefore tested the effect of tryptophan availability on the cell response to these compounds. For this, we first employed a *trp1*Δ deletant with disrupted cellular tryptophan biosynthesis and reliant on uptake of exogenous tryptophan. A sensitive trend for *trp1*Δ was observed in response to glabridin (Fig. 8A [*P* = 0.06]; Table S5; corresponding full growth curves in Fig. S7A and B) suggesting a defective capacity to rely on exogenous tryptophan (e.g., via Tat2 transport). We therefore used *TAT2*-overexpressing cells and/or tryptophan supplementation to investigate whether these effects could be rescued in glabridin-treated *trp1*Δ cells. Glabridin hypersensitivity in the *trp1*Δ deletant was partly rescued either by *TAT2* overexpression or tryptophan (T) supplementation (Fig. 8B). No further rescue was observed upon tryptophan supplementation when *TAT2* was overexpressed (Fig. S7C). These data support the hypothesis that glabridin impairs transmembrane tryptophan import.

### Role of protein biosynthesis and localization in response to wighteone.

Overrepresented gene functions mediating sensitivity (i.e., deletants were resistant) to wighteone were associated with cytoplasmic translation (i.e., protein biosynthesis) and ribosomal biogenesis (Fig. 3B). Almost all genes associated with the cytoplasmic translation GO term encoded ribosomal subunits (Table S7). This suggests that intact ribosomal function sensitizes the cells to wighteone. The GO enrichment analysis had also indicated ER to be the dominant cellular component of gene annotations where deletion produced sensitivity to wighteone (Table S8). More specifically, ER-related functions, such as the oligosaccharide-lipid intermediate biosynthetic process and the protein N-linked glycosylation (both related to mannoprotein biosynthesis), were overrepresented (Table S9).

Furthermore, from the 74 genes associated with cellular protein localization mediating resistance to wighteone (Fig. 3B), we could identify three clusters of highly sensitive deletants (GR ≥ 7.5) with genes encoding (i) proteins involved in protein sorting to various organelles, including Get1 and Get4, which function in the ER membrane (38), (ii) vacuolar proteases necessary for degradation of aberrant proteins synthesized in the ER (Pep4, Ape1), and (iii) alleviators of ER stress (Ire1) (relevant data summarized in Table S10). The above information implies that ER stress, typically associated with aberrances in protein synthesis or folding, may be a cellular response specifically to wighteone.

As an adaptive mechanism to ER stress, cells trigger the unfolded protein response (UPR). The deletant of the most conserved UPR marker, the ER transmembrane protein Ire1, scored highly sensitive to wighteone as described above (GR = 7.8) (Table S10). Furthermore, the deletants lacking genes encoding the vacuolar proteins Pep4 and Vtc4, which work in concert at the vacuolar membrane to degrade newly synthesized misfolded proteins coming from the ER (39–41), yielded some of the strongest wighteone hypersensitivity phenotypes in the screen data set (GR*_pep4_*_Δ_ = 22.0 and *P* = 5E−09 and GR*_vtc4_*_Δ_ = 48.0 and *P* = 4E−05) (Table S3). However, the deletant lacking Hac1, encoding a transcription factor regulated by Ire1 that mediates the activation of downstream UPR genes coding for chaperones, foldases, and lipid synthesis (42), did not exhibit sensitivity in the wighteone screen (Table S3) and, in dedicated growth tests with continuous shaking, proved significantly (*P* < 0.05) (Table S5) resistant specifically to wighteone (Fig. S8A and Fig. S9A and B). Similar to *hac1*Δ cells, the deletant strain for the cytoplasmic chaperone Hsp104, a disaggregase that rescues stress-damaged proteins from an aggregated state (43), was also significantly (*P* < 0.05) (Table S5) wighteone resistant (Fig. S8B and Fig. S9C and D).

Overall, deletants of several genes involved in yeast’s response to ER stress gave strong phenotypes exclusively in response to wighteone, as discussed further below.

## DISCUSSION

**FIG 9:**
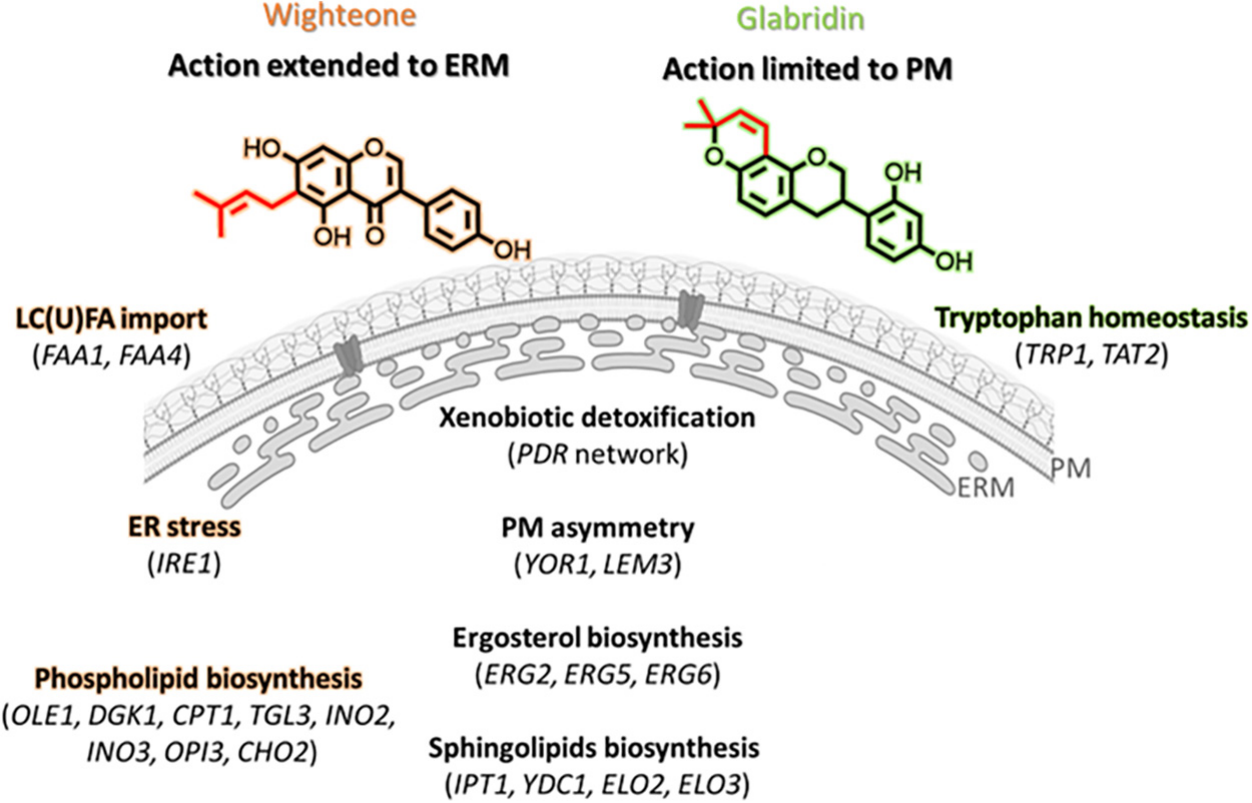
Overview of the most dominant and/or distinguishing cellular processes in food spoilage yeasts along with main associated genes that were either differentially expressed (transcriptomics) or whose deletion resulted in altered phenotype (chemogenomics) in the presence of prenylated isoflavonoids wighteone (highlighted in orange) and glabridin (highlighted in green). PM, plasma membrane; ERM, endoplasmic reticulum membrane.

The prenylated isoflavonoids wighteone and glabridin have shown potent antifungal activity against *Z. parabailii* (11). Here, we looked into the main cellular responses and targets of these antifungal compounds in food spoilage yeasts to shed light into their mechanism of action. From the transcriptomic analysis, we found that (xenobiotic) transmembrane transport was the most affected biological process in cells responding to the two prenylated isoflavonoids. Moreover, FA metabolism and lipid metabolism, together with proteostasis (after short exposure) and DNA replication (after longer exposure to prenylated isoflavonoids), were also affected. Interestingly, the apoptosis-related genes *MAC1* and *NUC1*, triggered by glabridin in the study by Moazeni et al. (16), were not deemed important in response to either of the two prenylated isoflavonoids in this study. The chemogenomic analysis showed that specific PM components such as sphingolipids and ergosterol and PM transporters influence the toxicity of both prenylated isoflavonoids. Interestingly, apparent impairment of tryptophan transmembrane transport was associated with glabridin’s toxicity (possibly due to a greater relative interaction of glabridin with the sphingolipid/ergosterol microdomains), whereas phospholipids and ER-related functions were found particularly to influence wighteone’s toxicity. Figure 9 illustrates a summary of the most dominant and/or distinguishing cellular effects in food spoilage yeast, found in this study, in response to prenylated isoflavonoids.

### Membrane lipids influence the toxicity of prenylated isoflavonoids to food spoilage yeast cells.

The yeast PM is composed of 70% phospholipids and equal amounts of sphingolipids and sterols (44). Prenylated isoflavonoids are known for their PM-disrupting properties in various microbial species (for examples, see references 11, 13, 28, and 45). We recently visualized the disruption of *Z. parabailli*’s PM by glabridin and wighteone, using transmission electron microscopy (TEM) (11). In the present study, *Z. parabailii* cells showed downregulation of processes such as FA β-oxidation (in response to glabridin and wighteone), as well as unsaturated fatty acid (UFA) biosynthesis and glycerol metabolism (only in response to wighteone), possibly related to (limited) biosynthesis of glycerophospholipids (Fig. 2). Furthermore, deletants of phospholipid biosynthesis in S. cerevisiae showed altered phenotypes (resistance and sensitivity), mainly in response to wighteone (Fig. 4). Altogether, the results suggest that both molecules disrupt the organization of PM phospholipids in yeasts, as they do in other microbial species, and that the distinct effects observed in wighteone-treated cells (i.e., ER-related effects) in this study may be related to an additional cellular target.

The chemogenomic data from S. cerevisiae together with independent assays carried out with *Z. parabailii* provided indications of roles of sphingolipids and ergosterol in the activity of the two prenylated isoflavonoids. The sensitivity to both compounds in *elo2*Δ S. cerevisiae cells (Fig. 5D), which accumulate reduced amounts of very-long-chain fatty acids (VLCFAs [C_20_ to C_26_]), main constituents of sphingolipids, suggests that sphingolipids limit the antimicrobial effects of prenylated isoflavonoids. The increased sensitivity of myriocin-preadapted *Z. parabailii* cells when treated with prenylated isoflavonoids (Fig. 6A and B) supports this finding. Toulmay et al. previously reported that C_26_ acyl chains help increase acyl chain packing density to prevent permeability by small molecules (46).

From the resistance phenotype of the *ipt1*Δ deletant (similarly observed for known membrane-disrupting agents) (47–49) and the hypersensitive *ydc1*Δ phenotype, both lacking enzymes of downstream sphingolipid biosynthesis, it seems that (the size and charge of) sphingolipids influenced the toxicity of prenylated isoflavonoids. More specifically, having the larger and more charged M(IP)_2_C (as in the *ydc1*Δ deletant) makes yeast cells more sensitive to the compounds than having more of the MIPC (as in the resistant *ipt1*Δ strain). The presence of M(IP)_2_C contributes to a looser packing of the hydrophobic membrane core, which may result in a more fluid membrane environment (50). From the above findings, it is suggested that the type of acyl chains (VLCFAs), together with the size and charge of the sphingolipid headgroup [M(IP)_2_C], modulates the resistance of yeast cells to prenylated isoflavonoids.

Apart from phospholipids and sphingolipids, ergosterol evidently limits the toxicity of prenylated isoflavonoids compared to its ergosterol intermediates, as evidenced by the sensitive deletants lacking downstream genes of ergosterol biosynthesis (Fig. 4). This is in line with hypersensitive *erg*Δ phenotypes observed with several small drugs (51–54). Nonetheless, due to the phenotype discrepancy between the *erg*Δ and the fluconazole-preadapted cells, further investigation is needed to elucidate relative actions of early versus late blocking of ergosterol biosynthesis.

Sphingolipids and ergosterol form highly ordered, tightly packed microdomains in PM in yeasts, acting as diffusion barriers (55–57), from which phospholipids are excluded (50, 58–62). The effects observed in strains with defective sphingolipid and ergosterol biosynthesis could due be either to altered transmembrane entry of prenylated isoflavonoids or disruption of membrane protein function necessary for the cell’s survival.

### Transmembrane (ATPase) phospholipid and tryptophan transporters influence the toxicity of prenylated isoflavonoids.

Besides PM lipids, PM transmembrane transporters were implicated in cellular responses to prenylated isoflavonoids. In particular, transmembrane ATPase transporters Yor1 and the protein homologous to S. cerevisiae Pdr5, involved in xenobiotic detoxification and proper phospholipid distribution across the PM (21, 63), were strongly upregulated by the two compounds (Fig. 2). Furthermore, the *yor1*Δ and *pdr5*Δ deletants, together with *lem3*Δ deletants lacking complementary function, yielded the most-sensitive phenotypes (see Table S6 in the supplemental material). Our dedicated growth tests (Fig. 7) highlighted that Yor1 and the Lem3-dependent flippase influence the toxicity of the two prenylated isoflavonoids. Khakhina et al. (63) detected similar phenotypes (i.e., *pdr5*Δ *yor1* resistance and *lem3*Δ sensitivity, as observed here) in response to the peptide aureobasidin A (AbA), an inhibitor of sphingolipid biosynthesis in the cytosol. It was proposed that the interaction of the floppases Yor1 and Pdr5 with the Lem3-dependent flippases alters the export of AbA (63) by regulating the function of an unknown PM AbA exporter. Based on the above data, it seems that PM floppases and flippases could play an important role in the toxicity of prenylated isoflavonoids either directly, by restoring the PM asymmetry possibly compromised by the compounds, or indirectly, by controlling PM protein function.

Furthermore, glabridin, but not wighteone, impaired tryptophan homeostasis, possibly through perturbation of Tat2 action (as evidenced by rescuing the sensitive *trp1*Δ strain through external tryptophan supplementation or *TAT2* overexpression) (Fig. 8B), which is in turn typically linked to membrane disruption (64–67). A recent integrative analysis of the yeast phenome, which refers to the aggregation, harmonization, and analysis of all published phenotypic screens of the S. cerevisiae knockout collection, highlighted that tryptophan homeostasis is a key point of resistance for over 1,000 chemical compounds (68). Exposure to glabridin also produced a greater relative sensitivity of the *lem3*Δ deletant than to wighteone (Fig. 7D; Fig. S6E and F). The Tat2 and Lem3 proteins are known to be localized in the same ergosterol/sphingolipid microdomains (64), suggesting that glabridin may interact more with these microdomains than wighteone. Glabridin is characterized by higher hydrophobicity, a 4-ring structure, and a more crooked backbone (absence of the C_2_-C_3_ unsaturated bond), than wighteone (Fig. 1). These features may enable better pi stacking, van der Waals interactions, and hydrogen bonding with ergosterol. These noncovalent interactions are also known to govern ergosterol’s interactions with the ergosterol-binding antifungals polyenes (69). This could result in specific disruption of the organization of the PM microenvironment that hosts Tat2 and Lem3. It should, however, be noted that a detrimental effect of glabridin on tryptophan import is unlikely to be a primary target of glabridin’s inhibitory MoA, since wild-type cells can meet cellular tryptophan demand by endogenous synthesis. These results would also imply that it is more likely the reduced diffusion posed by sphingolipids and ergosterol composing these microdomains, rather than the proper PM sorting of proteins in specific PM microdomains, that may limit the high toxicity of prenylated isoflavonoids.

### Wighteone may extend its primary action to the ER.

In addition to PM lipid and transporter proteins, prenylated isoflavonoids upregulated genes related to proteostasis (Fig. 2). For wighteone specifically, deletants of functions associated with protein biosynthesis and alleviation of ER stress contributed to resistance and sensitivity to wighteone, respectively (Table S10), suggesting that wighteone may disturb protein biosynthesis and exacerbate yeast susceptibility in the absence of relevant adaptive responses.

Ire1 is an inositol-requiring enzyme, embedded in the ER membrane, which is activated by two forms of ER stress: (i) misfolded proteins through its canonical sensing mechanism in the ER lumen (70) and (ii) defects in the ER membrane (ER lipid bilayer stress). We found that *ire1*Δ cells, together with *dgk1*Δ and *ice2*Δ cells lacking crucial factors for ER membrane biogenesis during ER stress, were highly sensitive (GR ≥ 7.5) to wighteone. The ER membrane is expanded as an adaptive response to ER stress, and this expansion is driven by phospholipid biosynthesis (71). In line with this, we observed *ino2*Δ, *ino4*Δ, and *opi3*Δ deletants, lacking major regulators of phospholipid biosynthesis (71–73), to be highly sensitive to wighteone (Fig. 4). Schuck et al. indicated that lipid biosynthesis-driven ER membrane expansion occurs without a concomitant increase in ER chaperone levels (like Hsp104) and is independent of Hac1, showing that ER expansion is not always coupled to the UPR (71). Based on the phenotypes observed in our study with *hac1*Δ and *hsp104*Δ (Fig. S8), we suggest that wighteone causes ER membrane stress, which is sensed by Ire1, without involving the UPR.

Defects in the ER membrane are sensed directly by Ire1’s cytosolic or transmembrane domain (70, 74, 75). Wiseman et al. (76) have shown through enzyme kinetic assays and cocrystallization studies that the nonprenylated flavonol quercetin potentiates the activation of Ire1 via a secondary binding site of its cytosolic domain. Wighteone shares structural similarities with quercetin (e.g., 7- and 4′- free hydroxyl groups and the presence of an unsaturated bond in C_2_-C_3_, as shown in Fig. 1), which may suggest similar intermolecular interactions with this secondary, cytosolic binding site of Ire1. Nonetheless, further investigation of wighteone’s potential binding to Ire1 is required.

Furthermore, in response to wighteone, we observed downregulation of glycerol catabolism and of FA desaturation (through downregulation of the ER-membrane bound, Ole1: i.e., the only source of endogenous LCUFAs in S. cerevisiae) (Fig. 2). This suggests a limited intracellular biosynthesis of glycerophospholipids in the ER in wighteone-treated cells. In addition, Faa1 and Faa4, known to translocate extracellular LC(U)FAs [in the case of blocked intracellular LC(U)FA biosynthesis], were found to underlie yeast’s sensitivity to wighteone (Fig. 5B and C [deletants were resistant]). This LC(U)FA translocation has been reported to occur through endocytosis (23). In line with this finding, we observed in our previous study signs of endocytosis specifically in wighteone-treated *Z. parabailii* cells (11). Upon translocation, activated LC(U)FAs are used either for lipid synthesis or for energy production in the peroxisome (22). Based on the sensitivity specifically to wighteone of deletants for peroxisomal import and degradation of LCFA functions (Table S11), it is inferred that normal peroxisomal function helps underpin the yeast’s resistance to this compound and that import of activated LC(U)FAs is mainly intended for lipid biosynthesis rather than for peroxisomal energy production.

In sum, the collective evidence suggests that wighteone may extend its action from the PM to the ER membrane (the latter known to contain relatively high quantities of LCUFA [22, 77–79]), causing ER membrane stress.

### Conclusions.

The prenylated isoflavonoids glabridin and wighteone are highly promising, novel, and natural antifungals. Yet, their distinct molecular properties together with the fact that membrane permeabilization is not always associated with killing (4, 11, 12) imply that the MoA of these prenylated isoflavonoids is not solely based on their PM activity, as has been traditionally believed. We employed transcriptomic sequencing, together with chemogenomic screening, of spoilage yeasts to describe the cellular responses and ultimately to better understand these agents’ modes of antifungal action. Lipid biosynthesis was found to be important for their toxicities. Sphingolipids and ergosterol, in particular, appeared to play roles in the inhibitory effects. This study also highlighted the importance of transmembrane ATPase transporters, including those involved in PM asymmetry, in the toxicity of the two prenylated isoflavonoids. The two compounds also showed some distinct effects. Collective evidence indicated that glabridin causes tryptophan starvation in deletants with disrupted tryptophan biosynthesis, which could be rescued by exogenous addition of tryptophan or by overexpression of the Tat2 transporter. Although genes involved in proteostasis, typically regulated in the ER, were upregulated in response to both compounds, deletants related to ER membrane stress responses showed sensitivity only to wighteone, suggesting the ER to be an additional target for wighteone. This study exemplifies the use of genomic tools combined with testing of arising hypotheses to shed new light on the antifungal action of prenylated isoflavonoids. Several new and well-supported hypotheses are presented here, providing clear directions for future research.

## MATERIALS AND METHODS

### Chemicals.

Glabridin (97%) was purchased from Wako Pure Chemical Industries, Ltd. (Osaka, Japan), and wighteone (96%) was purchased from Plantech UK (Reading, United Kingdom). Myriocin (≥95%) and fluconazole (≥98%) were supplied by Sanbio B.V. (Uden, the Netherlands). Stock solutions of the two prenylated isoflavonoids myriocin and fluconazole were prepared in dimethyl sulfoxide (DMSO) (Duchefa Biochemie, Harleem, the Netherlands). Components for YPD (1% [wt/vol] yeast extract, 2% bacto-peptone, 2% glucose) were purchased from Oxoid, Ltd. (Basingstoke, United Kingdom), unless stated otherwise. Sodium chloride and isopropanol were supplied by Fisher Scientific (Loughborough, United Kingdom). Sodium acetate, EDTA, glycerol, tryptophan, chloroform, Tri reagent, diethyl pyrocarbonate (DEPC), water, and glass beads (diameter of 425 to 600 μm) were supplied by Sigma-Aldrich (Dorset, United Kingdom). HCl was purchased from Honeywell Fluka (United Kingdom) and SDS from Melford Laboratories, Ltd. (Suffolk, United Kingdom).

### Transcriptomic response of *Z. parabailii* cells to wighteone and glabridin.

Starter cultures of Zygosaccharomyces parabailii ATCC 60483 (purchased from the American Type Culture Collection, Wessel, Germany) were grown in triplicate from single colonies overnight and diluted to an optical density at 600 nm (OD_600_) of ~0.1 in 600 mL of YPD broth (pH adjusted to 4.0 using 5 M HCl). Cultures were grown to the exponential phase (OD_600_ of ~2.0) at 30°C at 120 rpm and harvested by centrifugation (1,500 × *g*, 5 min). Cells were resuspended in 60 mL of YPD broth (pH 4) and split between six 50-mL tubes (six conditions per independent culture: one control and two with prenylated isoflavonoids, each at two time points). Tubes were supplemented with glabridin or wighteone to final concentrations of 20 and 8 μg/mL, respectively, having a mild effect on yeast survival after 120 min (>85% of survival according to CFU counts compared to the control) or DMSO solvent alone as control. Tubes were incubated at 30°C at 120 rpm for 30 min and 2 h. Cultures were harvested by centrifugation (1500 × *g*, 5 min), and RNA from half of the cells was extracted as explained below.

For RNA extraction, cells were resuspended in 500 μL of RNA extraction buffer (0.6 M sodium chloride, 0.2 M sodium acetate, 100 mM EDTA, 4% [wt/vol] SDS), together with glass beads (diameter of 425 to 600 μm). Samples were vortexed at maximum speed for 1 min. Liquid fractions were collected and transferred to new tubes. One milliliter of Tri reagent was added to the samples. Samples were mixed by inversion and incubated for 10 min at room temperature. Chloroform (200 μL) was added, and samples were vortexed before being incubated for 3 min at room temperature. Samples were centrifuged at 16,000 × *g* for 10 min. The upper aqueous phase was transferred to new tubes, and an equal volume of isopropanol was added. Samples were mixed by inversion and incubated for 20 min at −20°C. After centrifugation at 16,000 × *g* for 10 min, pellets were washed with 70% ethanol. Once the ethanol was entirely removed, the pellets were resuspended in 100 μL of DEPC-water. RNA was further cleaned up using the NucleoSpin RNA kit (Macherey-Nagel). RNA concentrations were measured using the Qubit fluorometer and the Qubit RNA BR assay kit (Thermo Fisher Scientific; Q10211) and RNA integrity was assessed using the Agilent TapeStation 4200 and the Agilent RNA ScreenTape assay kit (Agilent; 5067-5576 and 5067-5577).

Indexed sequencing libraries containing the 3′ ends of polyadenylated transcripts were prepared from 500 ng of total RNA using the QuantSeq 3′ mRNA-Seq library prep kit FWD for Illumina (Lexogen; 5001-5004) and the Lexogen i7 6-nt index set (Lexogen; 7001-7096). Libraries were quantified using the Qubit fluorometer and the Qubit double-stranded DNA (dsDNA) HS kit (Thermo Fisher Scientific; Q32854). Library fragment-length distributions were analyzed using the Agilent TapeStation 4200 and the Agilent high-sensitivity D1000 ScreenTape assay (Agilent; 5067-5584 and 5067-5585). Libraries were pooled in equimolar amounts, and final library quantification was performed using the KAPA library quantification kit for Illumina (Roche; KK4824). The library pool was sequenced on the Illumina NextSeq 500 on a NextSeq 500 high-output kit v2.5 75 cycle kit (Illumina; 20024906), to generate ~5 million 75-bp single-end reads per sample. Raw reads were trimmed using Cutadapt v.3.0 using the following parameters: -m 35 -a AGATCGGAAGAGCACACGTC –trim-n -a A{18} -a T{18} –nextseq-trim = 10. Trimmed reads were aligned to *Z. parabailii* ATCC 60483 (ASM198439v2) reference genome using Star v.2.7.6a with the following parameters: –outFilterType BySJout –outFilterMultimapNmax 20 –alignSJoverhangMin 8 –alignSJDBoverhangMin 1 –outFilterMismatchNmax 999 –outFilterMismatchNoverLmax 0.6 –alignIntronMin 20 –alignIntronMax 1000000 –alignMatesGapMax 1000000 –outSAMattributes NH HI NM MD. Aligned reads were counted using HTSeq v.0.12.4 using the following settings: -m intersection-nonempty -s yes -f bam -r pos. The obtained gene counts were further analyzed using the standard analysis protocol with DESeq2 1.30.1. Principal-component analysis (PCA) plots revealed a batch effect associated with one (R1) of the three biological replicate experiments used to produce RNA samples. To account for this, R1 was added to the design formula (R1 + condition).

Gene ontologies were obtained from quickGO (https://www.ebi.ac.uk/QuickGO/annotations). GO analysis was performed using the aspect Biological Process. The “lfchSrink” function from DESeq2 with type ashr was used to generate shrunken log_2_ fold change (FC). Genes were split into two groups based on whether they were upregulated or downregulated, and enrichment analysis was performed per group using R package topGO. The runTest function was applied with the weight01 algorithm and test fisher. Genes of interest were determined based on shrunken log_2_ FC of >1 and a Benjamini-Hochberg (BH) adjusted *P* value (*P*_adj_) of <0.1. Gene scores were calculated as shown in equation 1:

(1) $$\text{score}={\text{log}}_{\text{2}}\text{ FC}\times -\log_{10}(P_{\text{adj}})$$

The enrichment ratio of each GO category was calculated as the fraction of observed genes from each GO category from all observed genes divided by the fraction of expected genes from each GO category from the total genes in the genome. To reduce redundancy between enriched GO terms, the web server REVIGO was used (80). The species was set to S. cerevisiae S288C (559292), and the threshold for redundancy reduction was set at 0.7 (default). A default semantic similarity measure (SimRel) was used. The choice of the representative terms was made based on the enrichment ratio.

### Chemogenomics using an S. cerevisiae deletant library.

The full set of homozygous deletion strains was generated in the haploid Saccharomyces cerevisiae BY4741 background (*MAT***a** *his3*Δ1 *leu2*Δ0 *met15*Δ0 *ura3*Δ0), obtained from Euroscarf (Frankfurt, Germany). Strains were routinely stored at −80°C in a 384-well format, in YPD medium supplemented with 15% (vol/vol) glycerol. For experimental purposes, deletion strains were inoculated from the frozen stocks into YPD broth (pH 4.0) with a 384-pin tool, and the resultant inoculated microplates (Greiner Bio-One, Stonehouse, United Kingdom) were incubated at 30°C. After overnight incubation, cultures were inoculated again to fresh YPD broth at pH 4 (75 μL per well final volume), supplemented as specified with wighteone or glabridin from a stock solution in DMSO. Control incubations received the same DMSO addition as with the compounds (1% final concentration). Growth readings (*A*_600_) were recorded after static incubation for 24 h at 30°C using a BioTek Powerwave XS microplate spectrophotometer. Screens were performed with a moderately high concentration of each compound so that both resistant and sensitive deletants could be discerned (5 μg/mL glabridin and 2.25 μg/mL wighteone, showing ~47% and ~27% growth, respectively, versus the control condition) and a low concentration for discerning hypersensitive deletants (2.5 μg/mL glabridin and 1.125 μg/mL wighteone, showing no detectable growth inhibition). Each screen was done in three biological replicates.

Growth ratios (GRs) for each strain were calculated by dividing normalized *A*_600_ readings obtained under control conditions (minus wighteone or glabridin) by those for parallel compound-supplemented incubations. First, the background *A*_600_ of the medium alone (0.126) was subtracted. Then, each value was divided by a correction factor (*A*_600_ average of the plate holding that strain/*A*_600_ average of the entire set of plates) to correct for any plate-to-plate variation. All values of <0 were replaced by 0.001 to eliminate 0 denominators. Growth ratios were not calculated for slow-growing strains (*A*_600_ of <0.1 in untreated plates after 24 h), as small *A*_600_ differences have a disproportionately large effect on their growth ratios. The above procedures normalized the mean growth ratio for all strains to 1.0, with either wighteone or glabridin, enabling discrimination of sensitive or resistant strains (i.e., those deviating from the mean response) according to a GR of >2.0 or <0.5, respectively. Note that under the 2.25-μg/mL wighteone and 5-μg/mL glabridin conditions used, the mean growth effect (before normalization) of wighteone was a GR of ~3.7 and that of glabridin was a GR of ~2.1.

As described above, the enrichment ratio of each GO category was calculated for the sensitive or resistant gene sets, and the redundancy reduction between GO terms was performed.

### Corroboration of deletant phenotypes in S. cerevisiae.

Single colonies of the S. cerevisiae *his6*Δ strain (used here as the appropriate wild type [WT] background) and of deletants of interest, all isogenic with S. cerevisiae BY4743 (*MAT***a**/**α** *his3*Δ1/*his3*Δ1 *leu2*Δ0/*leu2*Δ0 *LYS2*/*lys2*Δ0 *met15*Δ0/*MET15 ura3*Δ0/*ura3*Δ0), were used to inoculate YPD broth cultures (pH 4.0) in Erlenmeyer flasks and incubated overnight at 30°C with orbital shaking at 120 rpm. Overnight cultures were diluted to an OD_600_ of 0.5 and cultured for a further 4 h in fresh medium. These 4-h mid/late-exponential cultures were diluted to an OD_600_ of 0.2, and 75-μL aliquots were transferred to 96-well microtiter plates (Greiner Bio-One, Stonehouse, United Kingdom) that had already received aliquots of 75 μL medium containing the antimicrobial dissolved in DMSO, such that 2% of the final 150-μL volume was DMSO, to give final antimicrobial concentrations as specified in Results. Plates were incubated at 30°C with shaking in a BioTek Powerwave XS microplate spectrophotometer, and the OD_600_ was recorded every 30 min. Growth assays of deletants were performed in three biological replicates (each performed in three technical replicates). The significance of the phenotypes was verified using two-tailed *t* tests where an equal variance was assumed (*P* < 0.05).

### Evaluation of tryptophan auxotrophy and rescue using tryptophan supplementation and/or TAT2 overexpression.

Tryptophan auxotrophy was evaluated with the S. cerevisiae *trp1*Δ deletant using the methodology described above.

Rescue of any phenotypes was done by external tryptophan supplementation and/or *TAT2* overexpression. Tryptophan was supplemented at a final concentration of 1 mM and was added at the same time as the antimicrobial (or DMSO alone as a control). Overexpression of the *TAT2* gene was achieved by transforming the background strain (BY4743) and the isogenic *his6*Δ and *trp1*Δ deletants with a pCM190 plasmid (empty vector) or pCM190 expressing the S. cerevisiae *TAT2* open reading frame (ORF) under Tet control, as produced previously (81). The lithium acetate method was used for transformation (82).

### Effect of lipid biosynthesis inhibitors on the toxicity of prenylated isoflavonoids against *Z. parabailii*.

*Z. parabailii* cells were streaked from a −80°C glycerol stock onto YPD (Brunschwig Chemie B.V., Amsterdam, the Netherlands) agar (VWR International B.V., Amsterdam, the Netherlands) and incubated for 48 h at 30°C. Stocks of lipid biosynthesis inhibitors myriocin (500 μg/mL) and fluconazole (2.5 mg/mL) were prepared in DMSO. Single colonies of *Z. parabailii* (ATCC 60483) were transferred to 10 mL YPD either nonsupplemented or supplemented with a final concentration of 0.2 μg/mL myriocin (83) (determined here as 1/5 MIC [Fig. S1A]) or 5 μg/mL fluconazole (determined here as 1/5 MIC [Fig. S1B**]**) and incubated for 18 h at 30°C. These overnight cultures were diluted either with nonsupplemented YPD or YPD supplemented with 1/5 MIC myriocin or fluconazole. (The final inoculum concentration of the myriocin-treated or untreated cells was on average 4.8 ± 0.5 log_10_ CFU/mL, and that of fluconazole-treated or untreated cells was 4.5 ± 0.1 log_10_ CFU/mL.) Stock solutions of the two prenylated compounds were prepared in DMSO and subsequently diluted with YPD (pH 4.0) that was either nonsupplemented or supplemented with the two lipid biosynthesis inhibitors. Equal volumes (100 μL) of yeast cells and prenylated isoflavonoid solutions [final test concentrations of prenylated (iso)flavonoids of 1.625 to 25 μg/mL, 1.0% [vol/vol] DMSO maximum concentration] were mixed into a 96-well plate. The OD_600_ was measured every 10 min with an Infinite 200 Pro M nanoplate reader (Tecan Benelux, B.V., the Netherlands) for 48 h at 30°C with high shaking. Negative controls (YPD suspension of yeasts with 1% DMSO) and blanks (compounds and YPD with and without lipid biosynthesis inhibitors) were used for optical comparison and sterility control. The experiments were performed in three biological replicates (each performed in technical duplicates).

### Change in time to detection.

To describe the growth curves, the parameter change in time to detection (ΔTTD) was employed for each relevant strain, as shown in equation 2:

(2) $${\text{ΔTTD}}_{i}={\text{TTD}}_{\text{treated}}-{\text{TTD}}_{\text{untreated}}$$

TTD is defined as the time taken for the OD to increase by 0.05 U from the start of the culture, *i* refers to the particular strain tested, TTD_treated_ is the time to detection of the culture *i* in the presence of the antimicrobial, and TTD_untreated_ is the time to detection of the control culture *i* in the absence of the antimicrobial.

### Data availability.

The complete RNA sequence data have been deposited in the ENA database under accession no. PRJEB55803.
