# Supplementary material for: Cellular Responses and Targets in Food Spoilage Yeasts Exposed to Antifungal Prenylated Isoflavonoids
Source: Microbiol Spectr. 2023 Jul 10;11(4):e01327-23. doi: 10.1128/spectrum.01327-23 (PMC10433819; doi:10.1128/spectrum.01327-23)
Supplement: Supplemental file 7 — Supplemental material. Download spectrum.01327-23-s0007.docx, DOCX file, 14.4 MB [file spectrum.01327-23-s0007.docx]

## Supplementary information

# Cellular responses and targets in food-spoilage yeasts exposed to antifungal prenylated isoflavonoids

Sylvia Kalli^a^, Cindy Vallieres^b^, Joseph Violet^b^, Jan-Willem Sanders^c^, John Chapman^c^, Jean-Paul Vincken^a^, Simon V. Avery^b^ and Carla Araya-Cloutier^a^

**Table S1A-C**. Differentially regulated genes (log 2-fold change, p-value and description) in *Z. parabailii* cells treated with prenylated isoflavonoids (transcriptomics).

 ****

**Table S2**. Genes contributing to GO terms, transcriptomics.

**Table S3**. Full deletant library used for chemogenomic screening.

**Table S4**. Growth ratio (GR) of key deletants of lipid biosynthesis-related functions, in response to wighteone and glabridin, obtained during chemogenomic screening. Deletants with a GR > 2.0 are considered sensitive, GR ≤ 0.5 are considered resistant). LCFA, long chain FAs. Deletants whose GR was not (significantly) altered (0.5 < GR ≤ 2.0 and/or p-value < 0.05) are shown with a (-). Phenotype data are derived from the *S. cerevisiae* deletant collection screens.

|  |  | **Wighteone** | | **Glabridin** | |
| --- | --- | --- | --- | --- | --- |
|  | **Deletant** | **GR** | **p-value** | **GR** | **p-value** |
| LCFA (C_12_–C_18_) import and activation | *faa1*Δ | - | - | 0.5 | 5 E-03 |
|  | *faa4*Δ | 0.3 | 6 E-04 | 0.5 | 1 E-03 |
| Sphingolipid biosynthesis | *ipt1*Δ | 0.2 | 4 E-03 | 0.5 | 2 E-04 |
|  | *ydc1*Δ | 29.8 | 8 E-07 | 39.0 | 7 E-07 |
|  | *elo1*Δ | 0.4 | 2 E-03 | - | - |
|  | *elo2*Δ | 3.9 | 3 E-03 | 5.3 | 2 E-04 |
|  | *elo3*Δ | 3.6 | 3 E-03 | 2.5 | 3 E-03 |
| Ergosterol biosynthesis | *erg2*Δ | 18.7 | 1 E-05 | 13.5 | 9 E-06 |
|  | *erg5*Δ | 8.5 | 4 E-05 | 7.4 | 2 E-05 |
|  | *erg6*Δ | 6.7 | 5 E-04 | 29.7 | 1 E-04 |
|  | *erg24*Δ | 2.1 | 2 E-02 | - | - |
| Phospholipid biosynthesis | *ale1*Δ | 0.1 | 3 E-02 | 0.3 | 2 E-02 |
|  | *cho2*Δ | 2.3 | 3 E-03 | - | - |
|  | *cpt1*Δ | 5.1 | 1 E-03 | 2.3 | 7 E-03 |
|  | *dgk1*Δ (*hsd1*Δ) | 28.3 | 3 E-06 | - | - |
|  | *dpp1*Δ | 2.1 | 5 E-03 | - | - |
|  | *ept1*Δ | 0.5 | 2 E-03 | - | - |
|  | *ino2*Δ | 4.4 | 4 E-05 | - | - |
|  | *ino4*Δ | 4.9 | 1 E-04 | - | - |
|  | *lpp1*Δ | 2.1 | 2 E-02 | - | - |
|  | *opi3*Δ | 2.7 | 3 E-05 | - | - |
|  | *pah1*Δ | 2.0 | 5 E-04 | - | - |
|  | *pct1*Δ | 0.5 | 3 E-05 | - | - |
|  | *tgl3*Δ | 4.7 | 6 E-06 | - | - |

**Table S5**. Growth curves (raw data) and significance of the corroborated phenotypes.

**Table S6**. Sensitivities of deletants of ABC plasma membrane transporters in response to wighteone and glabridin obtained during chemogenomic screening. Deletants with a GR > 2.0 are considered sensitive and those with a GR ≤ 0.5 are considered resistant. Deletants whose GR was not (significantly) altered (0.5 < GR ≤ 2.0 and/or p-value < 0.05) are shown with a (-). Data is taken from **Table S3**.

| **ABC plasma membrane transporters** | **Deletant** | **Wighteone** | | **Glabridin** | |
| --- | --- | --- | --- | --- | --- |
|  |  | **GR** | **p-value** | **GR** | **p-value** |
|  | *yor1*Δ | 8.1 | 7 E-06 | - | - |
|  | *pdr5*Δ | 9.0 | 4 E-05 | 18.9 | 2 E-05 |
|  | *pdr12*Δ | 5.4 | 3 E-05 | - | - |
|  | *pdr15*Δ | 4.6 | 8 E-06 | - | - |
|  | *pdr18*Δ | 2.9 | 2 E-02 | - | - |
|  | *snq2*Δ | 2.0 | 5 E-02 | - | - |
|  | *adp1*Δ | 2.3 | 2 E-03 | - | - |

**Table S7.** Cytoplasmic translation-associated genes of *S. cerevisiae* conferring resistance to wighteone.

| **GO term** | **Description** | **p-value** | **Enrichment (N,B, n, b)** | **Genes** |
| --- | --- | --- | --- | --- |
| GO:0002181 | Cytoplasmic translation | 5 E-06 | 2.4 (3872, 89, 511, 28) | RPP1b |
|  |  |  |  | TMA19 |
|  |  |  |  | RPL14A |
|  |  |  |  | RPL17B |
|  |  |  |  | RPS26B |
|  |  |  |  | RPL36A |
|  |  |  |  | RPL26B |
|  |  |  |  | RPS28B |
|  |  |  |  | RPL40A |
|  |  |  |  | RPL38 |
|  |  |  |  | RPL41B |
|  |  |  |  | RPS16A |
|  |  |  |  | RPS11B |
|  |  |  |  | RPS7B |
|  |  |  |  | RPS14B |
|  |  |  |  | RPL16A |
|  |  |  |  | RPL15B |
|  |  |  |  | RPS21A |
|  |  |  |  | RPS22B |
|  |  |  |  | RPS6A |
|  |  |  |  | RPP2B |
|  |  |  |  | RPL43A |
|  |  |  |  | RPS29B |
|  |  |  |  | RPL33B |
|  |  |  |  | RPL11B |
|  |  |  |  | RPS7A |
|  |  |  |  | RPS24A |
|  |  |  |  | RPL21B |

**Table S8**. GO enrichment analysis for cellular components of gene annotations where deletion produced sensitivity (or resistance only in the case of wighteone) to prenylated isoflavonoids.

|  | **GO term** | **Description** | **p-value** | **Enrichment ratio** | **Number of significant genes** |
| --- | --- | --- | --- | --- | --- |
| **Wighteone** |  |  |  |  |  |
| Sensitive | GO:0005789 | endoplasmic reticulum membrane | 3 E-05 | 1.6 | 65 |
|  | GO:0005783 | endoplasmic reticulum | 4 E-05 | 1.4 | 112 |
|  | GO:0044432 | endoplasmic reticulum part | 5 E-05 | 1.5 | 71 |
|  | GO:0016021 | integral component of membrane | 5 E-05 | 1.3 | 195 |
|  | GO:0031224 | instinsic component of membrane | 2 E-04 | 1.2 | 202 |
|  | GO:0044425 | membrane part | 9 E-04 | 1.2 | 234 |
|  | GO:0034399 | nuclear periphery | 3 E-04 | 4.4 | 8 |
| Resistant | GO:0044445 | Cytosol | 4 E-06 | 2.2 | 33 |
|  | GO:0022625 | Cytosolic large ribosomal subunit | 2 E-04 | 2.6 | 15 |
|  | GO:0032991 | Protein-containing complex | 3 E-04 | 1.2 | 181 |
|  | GO:0005737 | Cytoplasm | 1 E-03 | 1.2 | 256 |
| **Glabridin** |  |  |  |  |  |
| Sensitive | GO:0044446 | intracellular organelle part | 2 E-03 | 1.5 | 79 |
|  | GO:0044422 | organelle part | 1 E-03 | 1.5 | 79 |
|  | GO:0044424 | intracellular part | 2 E-02 | 1.1 | 116 |
|  | GO:0044445 | cytosolic part | 1 E-02 | 3.6 | 12 |
|  | GO:0044444 | cytoplasmic part | 5 E-02 | 1.2 | 86 |
|  | GO:0005789 | endoplasmic reticulum membrane | 5 E-03 | 2.4 | 20 |
|  | GO: 0005783 | endoplasmic reticulum | 5 E-03 | 2.2 | 30 |
|  | GO:0044432 | endoplasmic reticulum part | 2 E-02 | 2.5 | 20 |
|  | GO:0022627 | cytosolic small ribosomal subunit | 2 E-02 | 5.7 | 7 |
|  | GO:0015935 | small ribosomal subunit | 3 E-02 | 4.2 | 8 |
|  | GO:0030684 | preribosome | 4 E-02 | 4.8 | 7 |
|  | GO:1990726 | Lsm1-7-Pat1 complex | 1 E-02 | 24.9 | 3 |
|  | GO:0070847 | core mediator complex | 5 E-02 | 14.2 | 3 |
|  | GO:0032991 | protein-containing complex | 2 E-02 | 1.5 | 52 |
|  | GO:0016592 | mediator complex | 2 E-02 | 12.0 | 4 |
|  | GO:0044464 | cell part | 3 E-02 | 1.1 | 117 |

**Table S9.** Selected over-represented GO functions associated with genes conferring resistance to wighteone.

| **GO term** | **Description** | **p-value** | **Enrichment (N,B, n, b)** | **Genes** |
| --- | --- | --- | --- | --- |
| GO:0006490 | oligosaccharide-lipid intermediate biosynthetic process | 5 E-06 | 14.5 (3827,7,191,5) | ALG8  ALG6  ALG12  ALG3 |
|  |  |  |  |  |
| GO:0006487 | protein N-linked glycosylation | 3 E-04 | 5.0 (3827,28,191,7) | AlG8  OST3  ALG6  MNN10  ALG12  DIE2  OST6 |
|  |  |  |  |  |
|  |  |  |  |  |
|  |  |  |  |  |
| GO:0045737 | positive regulation of cyclin-dependent protein serine/threonine activity | 5 E-04 | 15.2 (3827,4,191,3) | YSP1  CLB2  SIP3 |

**Table S10.** Wighteone hyper-sensitive deletants (GR ≥ 7.5) associated with the cellular protein localization GO term. Data on glabridin are presented for comparison. Deletants whose GR was not (significantly) altered (0.5 < GR ≤ 2.0 and/or p-value < 0.05) are shown with a (-).

| **Cellular protein localization** | | | | | |
| --- | --- | --- | --- | --- | --- |
|  | **Wighteone** | | **Glabridin** | |  |
| **Deletant** | **GR** | **p-value** | **GR** | **p-value** | **(Short) gene description** |
| *syh1*Δ | 23.8 | 1E-04 | 3.9 | 1 E-03 | Protein of unknown function that influences nuclear pore distribution |
| *cos10*Δ | 23.7 | 2 E-05 | 10.9 | 1 E-05 | Endosomal protein involved in turnover PM proteins |
| *pep4*Δ | 22.4 | 5 E-09 | - | - | Vacuolar aspartyl protease (proteinase A) |
| *slm1*Δ | 16.2 | 9 E-06 | 3.1 | 5 E-07 | Phosphoinositide PI4,5P(2) binding protein |
| *ice2*Δ | 15.5 | 2 E-05 | 2.3 | 2 E-07 | Integral ER membrane protein; promotes ER membrane expansion |
| *slk19*Δ | 15.2 | 2 E-05 | - | - | Kinetochore-associated protein; required for chromosome segregation and kinetochore clustering |
| *tim21*Δ | 14.9 | 6 E-06 | 3.7 | 8 E-04 | Interacts with the TOM complex and with respiratory enzymes |
| *yet1*Δ | 14.8 | 3 E-06 | - | - | ER transmembrane protein; may interact with ribosomes |
| *emp70*Δ | 14.8 | 4 E-06 | - | - | Protein with a role in cellular adhesion and filamentous growth; also endosome-to-vacuole sorting |
| *gsf2*Δ | 14.3 | 4 E-07 | 6.2 | 4 E-06 | ER localized integral membrane protein; may promote secretion of certain hexose transporters, including Gal2p; involved in glucose-dependent repression |
| *vps70*Δ | 13.4 | 7 E-05 | 9.6 | 5 E-05 | Protein of unknown function involved in vacuolar protein sorting |
| *nce102*Δ | 13.1 | 6 E-06 | 10.2 | 8 E-07 | Protein of unknown function; contains transmembrane domains; involved in secretion of proteins that lack classical secretory signal sequences; component of the DGIs |
| *ape1*Δ | 12.5 | 2 E-06 | - | - | Vacuolar aminopeptidase yscI |
| *mog1*Δ | 9.8 | 3 E-03 | 27.3 | 2 E-04 | Conserved nuclear protein that interacts with GTP-Gsp1p; stimulates nucleotide release from Gsp1p |
| *ynr065C*Δ | 9.7 | 8 E-06 | - | - | Protein of unknown function; possible role in actin patch formation |
| *pex12*Δ | 9.6 | 8 E-06 | - | - | C3HC4-type RING-finger peroxin and E3 ubiquitin ligase; required for peroxisome biogenesis and peroxisomal matrix protein import |
| *kap120*Δ | 9.6 | 4 E-06 | - | - | Karyopherin responsible for the nuclear import of Rpf1p |
| *ylh47*Δ | 9.2 | 2 E-05 | - | - | Mitochondrial inner membrane protein |
| *ras1*Δ | 8.3 | 3 E-04 | - | - | GTPase involved in G-protein signaling in adenylate cyclase activation; localized to the PM |
| *rpd3*Δ | 8.0 | 4 E-05 | - | - | Histone deacetylase; regulates transcription, silencing, autophagy |
| *rax2*Δ | 7.9 | 4 E-05 | - | - | Protein required for the maintenance of bud site selection |
| *get4*Δ | 7.8 | 2 E-04 | - | - | Protein involved in inserting tail-anchored proteins into ER membranes |
| *ire1*Δ | 7.8 | 7 E-05 | - | - | Serine-threonine kinase and endoribonuclease; transmembrane protein that mediates UPR by regulating Hac1p synthesis through HAC1 mRNA splicing |
| *hls1*Δ | 7.7 | 3 E-05 | - | - | Nim1p-related protein kinase; septin-binding kinase that localizes to the bud neck septin ring and regulates the morphogenesis checkpoint |
| *get1*Δ | 7.6 | 1 E-04 | - | - | Involved in insertion of proteins into the ER membrane |
| *csr2*Δ | 7.5 | 6 E-06 | - | - | Nuclear ubiquitin protein ligase binding protein; may regulate utilization of nonfermentable carbon sources and endocytosis of plasma membrane proteins |
| *elm1*Δ | 7.5 | 2 E-05 | - | - | Serine/threonine protein kinase; regulates the orientation checkpoint, the morphogenesis checkpoint and the metabolic switch from fermentative to oxidative metabolism |
| *vac8*Δ | 7.5 | 4 E-05 | 12.9 | 4 E-08 | Vacuole-specific Myo2p receptor |

TOM: Translocase of the Outer Mitochondrial Membrane; UPR: Unfolded Protein Response; DIGs: detergent-insoluble glycolipid-enriched complexes.

**Table S11**. GR of deletants of peroxisome function, biogenesis and maintenance in response to the prenylated isoflavonoids, wighteone and glabridin. Deletants with a GR > 2.0 are considered sensitive and those with GR ≤ 0.5 are considered resistant. Deletants whose GR was not (significantly) altered (0.5 < GR ≤ 2.0 and/or p-value < 0.05) are shown with a (-).

| **Peroxisome** | **Deletant** | **Wighteone** | | **Glabridin** | |
| --- | --- | --- | --- | --- | --- |
|  |  | **GR** | **p-value** | **GR** | **p-value** |
| Peroxisomal transporter complex importing LCFAs | *pxa1*Δ | 11.1 | 5 E-05 | - | - |
| β-oxidation pathway | *pxo1*Δ | 3.3 | 3 E-03 | 2.1 | 6 E-03 |
|  | *pot1*Δ | 2.8 | 1 E-04 | - | - |
| Peroxisomal docking/translocation | *pex3*Δ | 2.4 | 3 E-03 | - | - |
|  | *pex10*Δ | 6.4 | 8 E-05 | 2.2 | 4 E-03 |
|  | *pex12*Δ | 9.6 | 8 E-06 | - | - |
|  | *pex13*Δ | 9.4 | 2 E-07 | - | - |
|  | *pex17*Δ | 6.5 | 6 E-05 | - | - |
|  | *pex19*Δ | 4.6 | 9 E-06 | - | - |
| Shuttling receptors/transporters of peroxisomal matrix proteins | *pex4*Δ | 2.4 | 4 E-03 | - | - |
|  | *pex5*Δ | 3.5 | 2 E-04 | - | - |
|  | *pex6*Δ | 6.8 | 3 E-04 | - | - |
|  | *pex7*Δ | 4.5 | 5 E-04 | - | - |
|  | *pex15*Δ | 3.2 | 2 E-04 | - | - |
| Regulation of peroxisome biogenesis, size and maintenance | *pex11*Δ | 0.2 | 3 E-02 | 0.4 | 0.03 |
|  | *pex25*Δ | 0.2 | 8 E-03 | - | - |
|  | *pex27*Δ | 2.1 | 0.01 | - | - |
|  | *pex29*Δ | 0.2 | 2 E-03 | - | - |
|  | *pex30*Δ | 0.5 | 4 E-04 | - | - |
|  | *pex31*Δ | 4.3 | 6 E-04 | - | - |
|  | *pex32*Δ | 7.7 | 2 E-05 | - | - |
|  | *pex34*Δ | 3.5 | 2 E-06 | - | - |


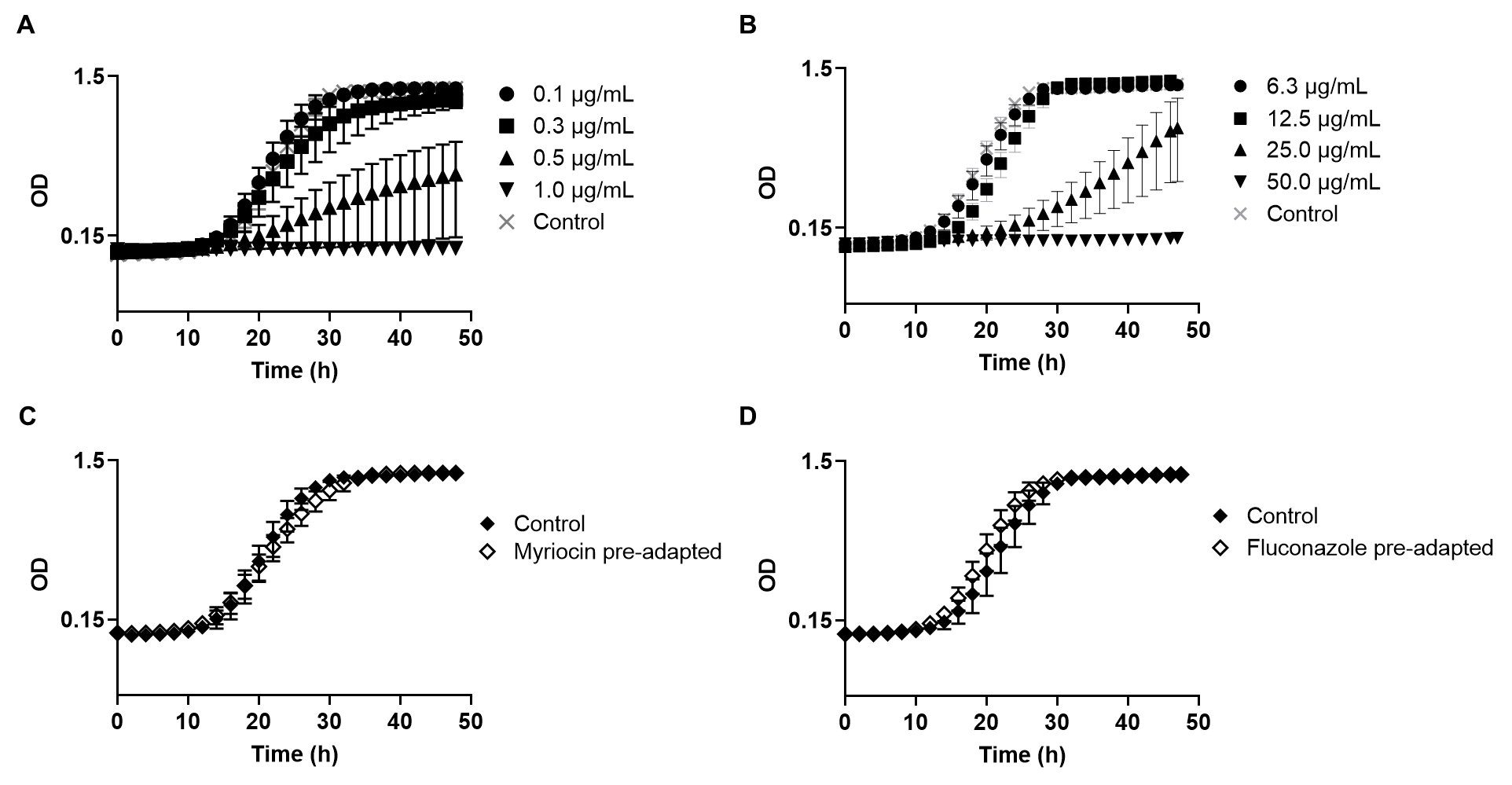


**Figure S1**. Determination of the minimum inhibitory concentration (MIC) of myriocin (**A**) and fluconazole (**B**) against *Z. parabailii* cells (ATCC 60483) and of the influence of YPD supplementation with 0.2 µg/mL myriocin (**C**) or 5 µg/mL fluconazole (**D**) on the yeast growth. Values shown are the means ± SEM of two (**A**-**B**) and three (**C**-**D**) biological replicates (each of which is the result of 2 technical replicates).


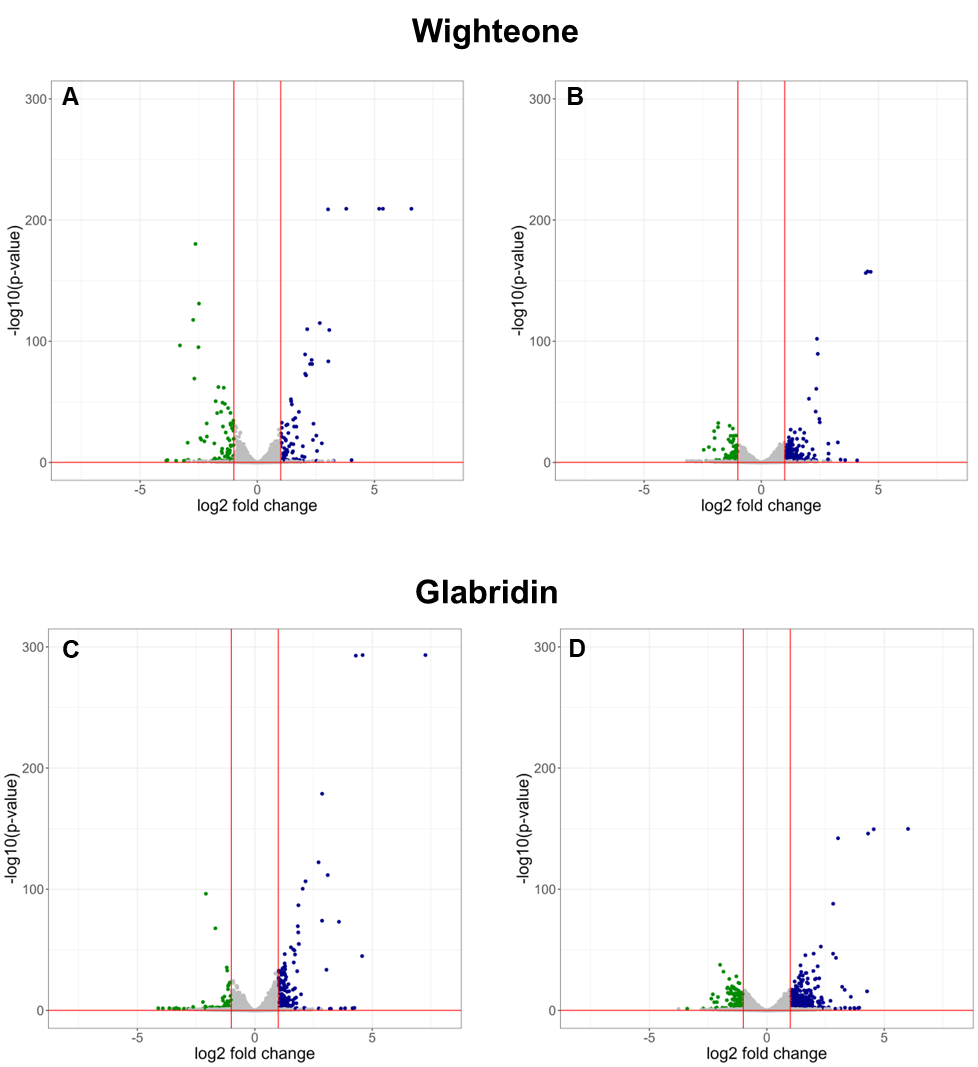


**Figure S2.** Volcano plots of *Z. parabailii* genes upon exposure to wighteone at 30 and 120 min (**A** and **B**) and glabridin at 30 and 120 min (**C** and **D**). Blue and green data points show significantly (p-value < 0.05) up- and down-regulated genes, respectively, with a log 2-fold change > |1.0|. Grey data points indicate non-significantly expressed genes with p-value > 0.05 and/or log 2-fold change < |1.0|.


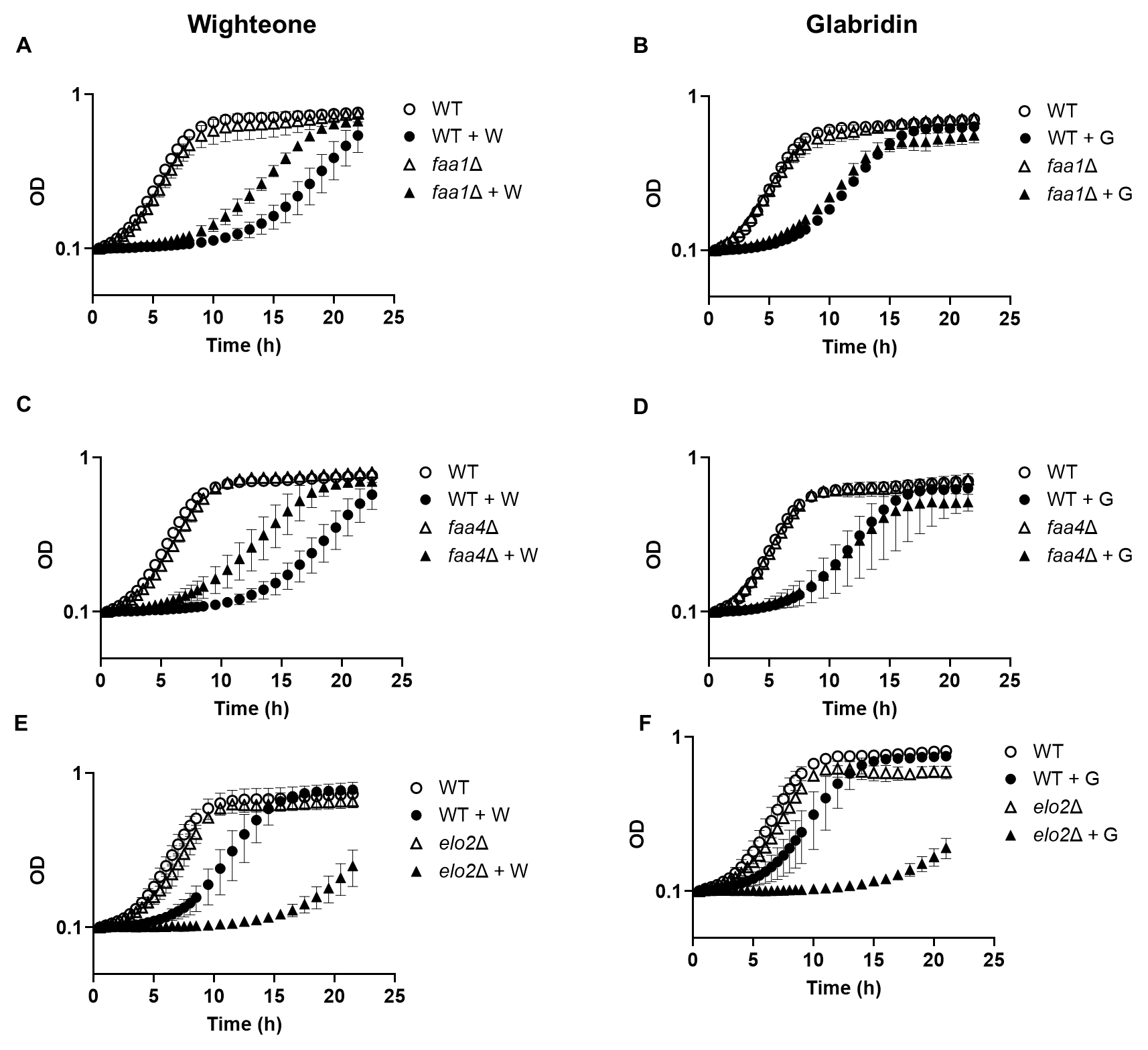


**Figure S3.** Full growth curves showing the resistance of mutants lacking the genes encoding for the fatty acyl-CoA synthetases, *FAA1* and *FAA4*, and sensitivity of mutants lacking the FA elongase Elo2 to wighteone (W) and glabridin (G). Treated *S. cerevisiae* WT, *faa1*Δ (**A**, **B**) and *faa4*Δ (**C**, **D**) strains were cultured in the presence of 5.0 μg/mL wighteone (**A**, **C**) or 10.0 μg/mL glabridin (**B**, **D**), whereas treated *elo2*Δ strains were cultured in the presence of 3.8 μg/mL wighteone (**E**) or 7.5 μg/mL glabridin (**F**). Values shown are the means ± SD of three biological replicates (each of which is the result of three technical replicates).


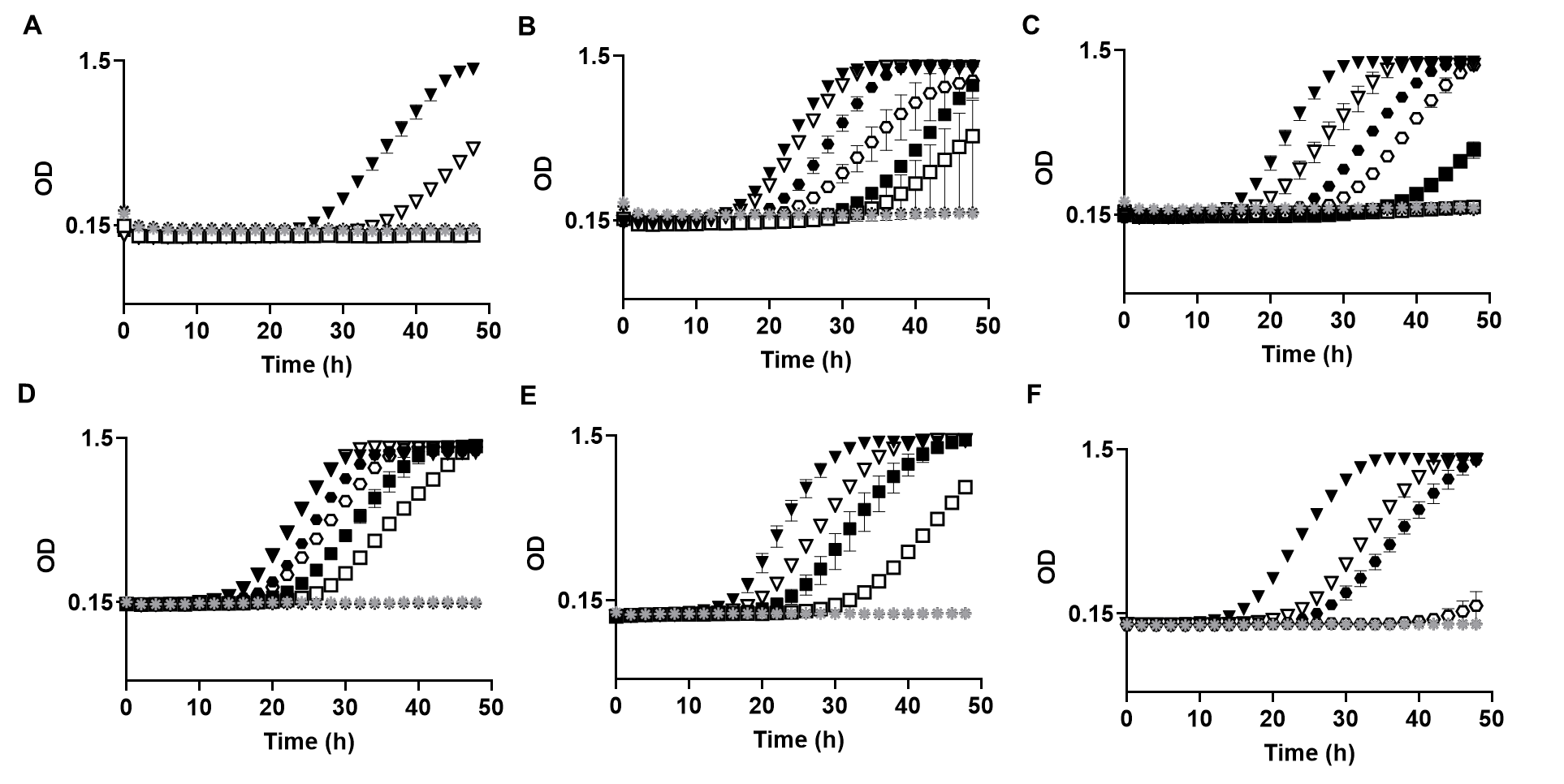


**Figure S4.** Three biological replicates showing the effect of myriocin-overnight treatment of *Z. parabailii* (ATCC 60483) cells exposed to sublethal concentrations of wighteone (**A-C**) and glabridin (**D**-**E**). Triangles, hexagons, squares, and asterisks represent a prenylated isoflavonoid concentration of 6.3, 9.4, 12.5 and 25.0 µg/mL, respectively. Open symbols (and grey asterisks) indicate cells treated with the inhibitor and filled symbols indicate control cells.


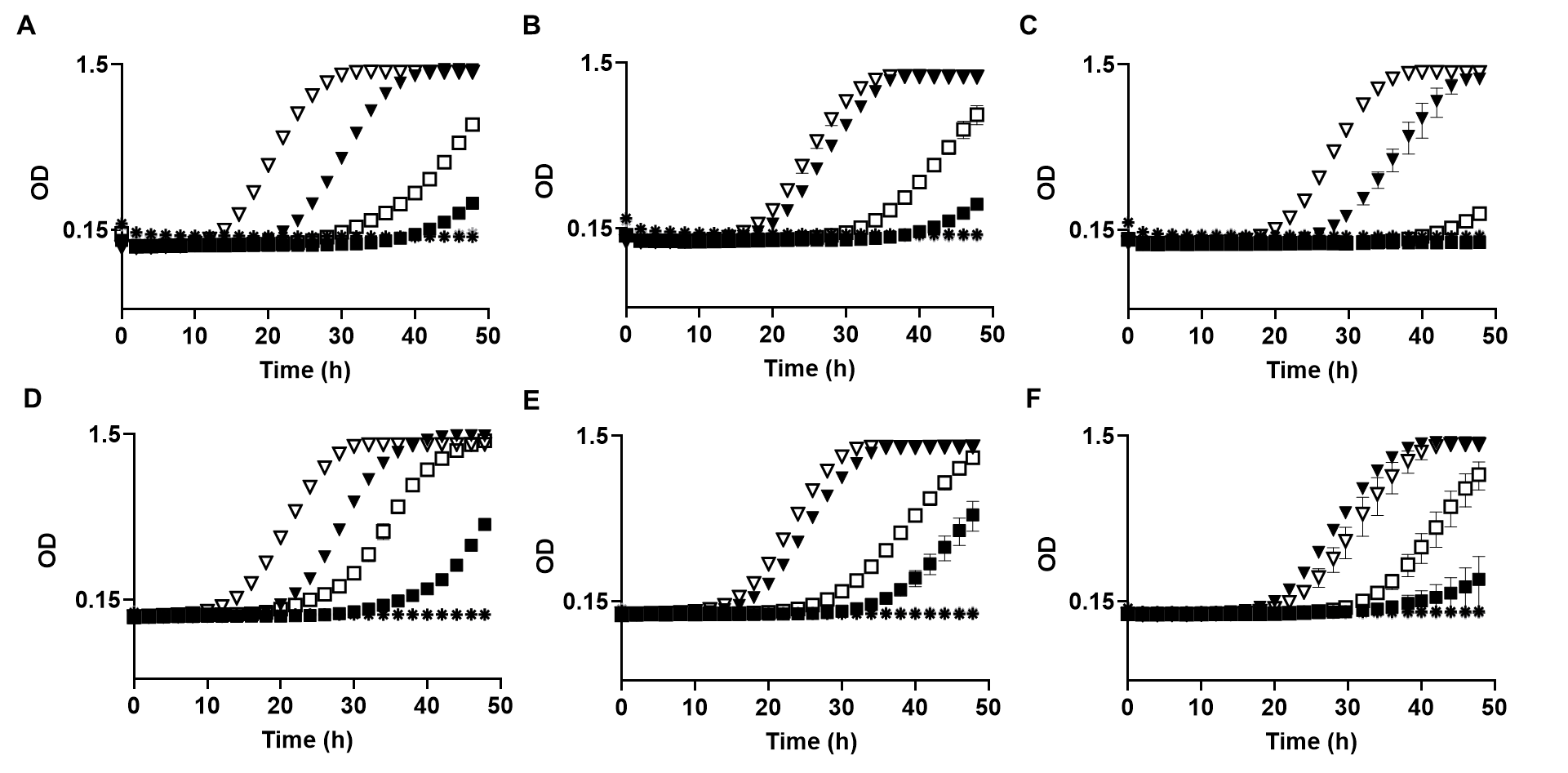


**Figure S5.** Three biological replicates showing the effect of fluconazole-overnight treatment of *Z. parabailii* (ATCC 60483) cells exposed to sublethal concentrations of wighteone (**A-C**) and glabridin (**D-E**). Triangles, squares and asterisks represent a prenylated isoflavonoid concentration of 6.3, 12.5 and 25.0 µg/mL, respectively. Open symbols (and grey asterisks) indicate cells treated with the inhibitor and filled symbols indicate control cells.


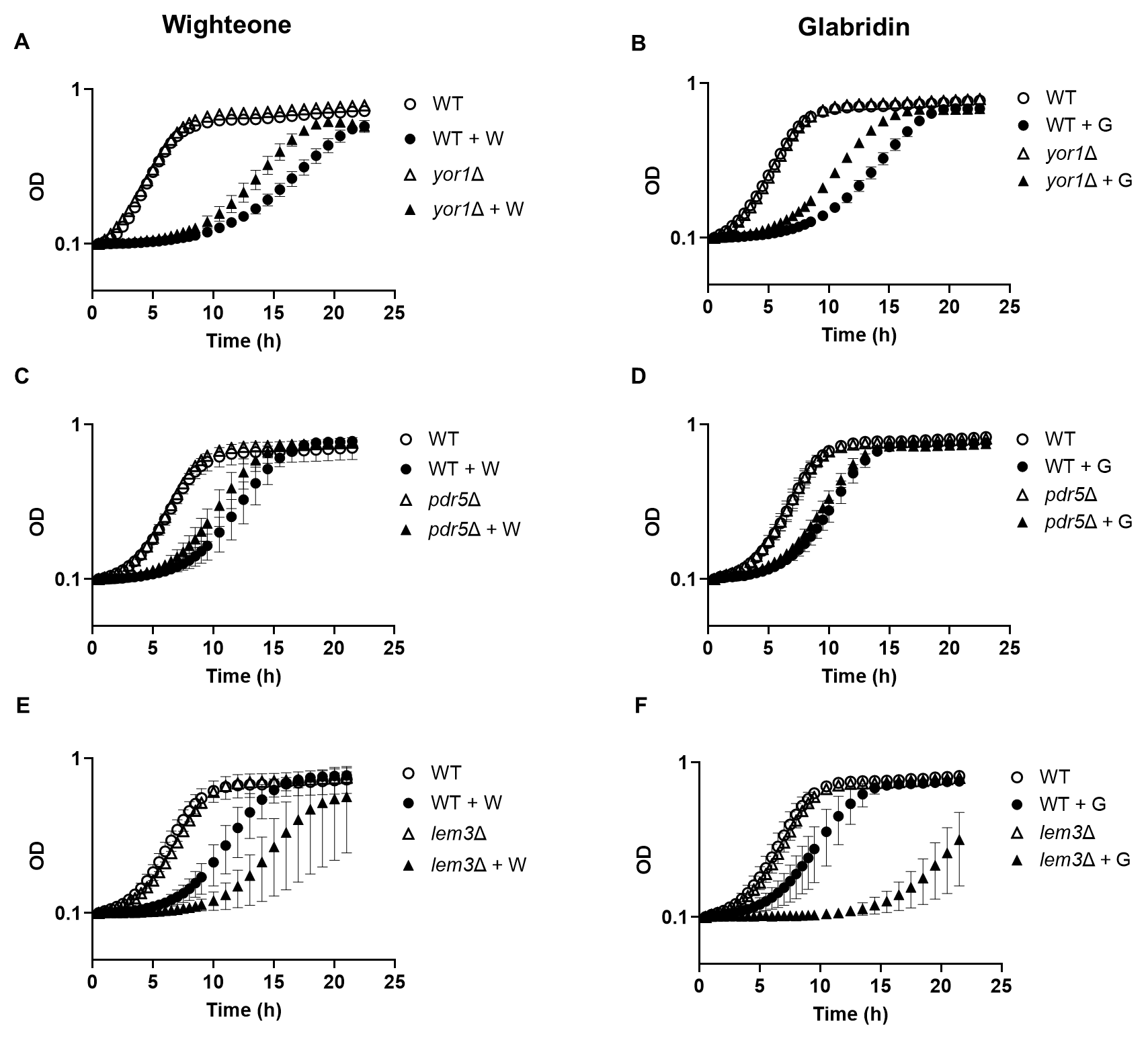


**Figure S6**. Full growth curves showing the sensitivity and resistance of *yor1*Δ and *lem3*Δ, respectively, to the prenylated isoflavonoids, wighteone, (W) and glabridin (G). Treated *S. cerevisiae* WT, *yor1*Δ, *pdr5*Δ strains were cultured in the presence of 3.8 μg/mL wighteone (**A**, **C**) or 7.5 μg/mL glabridin (**B**, **D**) and the treated *lem3*Δ strain was cultivated in the presence of 5.0 μg/mL wighteone (**E**) or 10.0 μg/mL glabridin (**F**). Values shown are the means ± SD of three biological replicates (each of which is the result of three technical replicates).


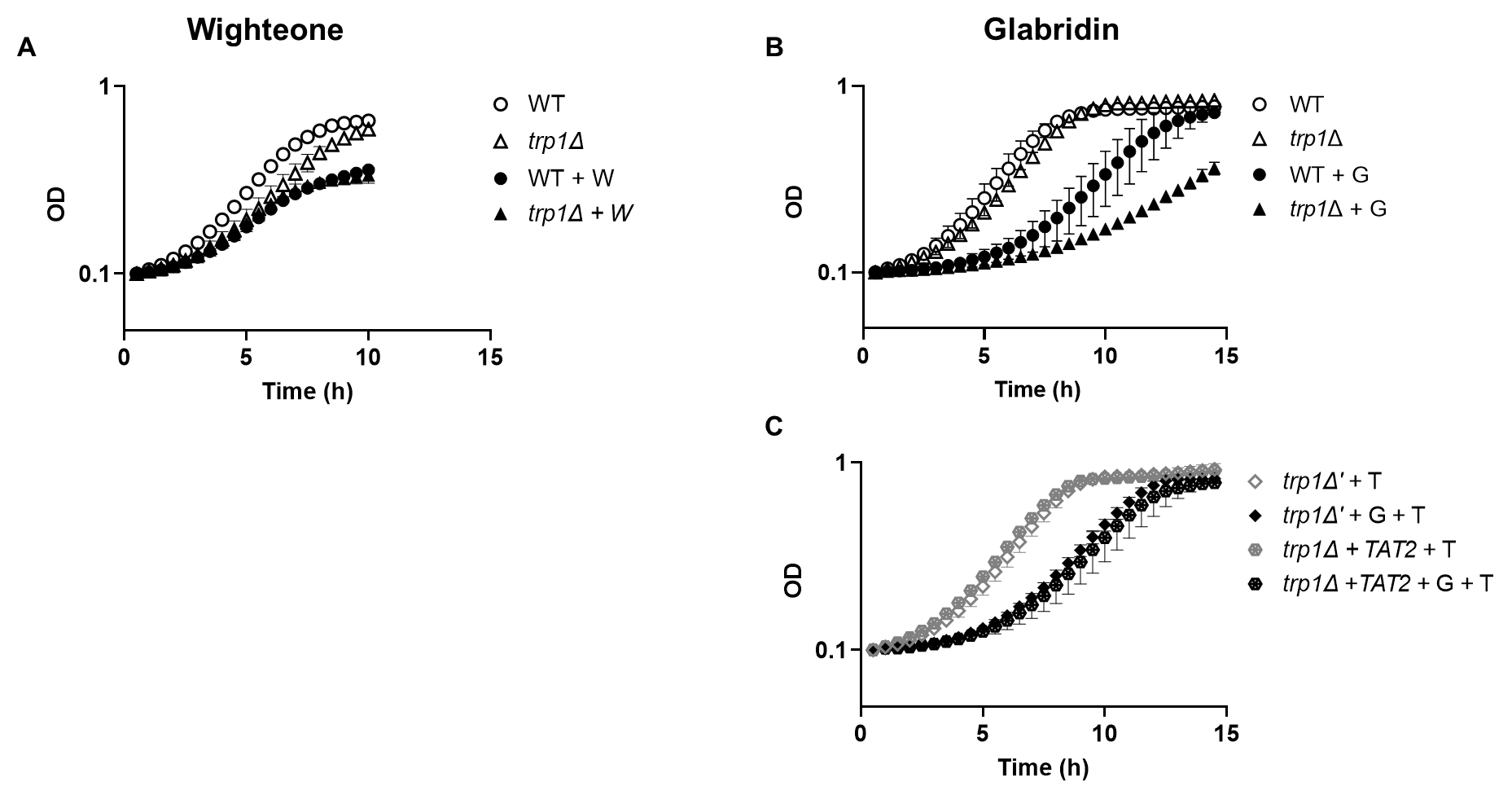


**Figure S7**. Full growth curves showing the sensitivity of *trp1*Δ cells to glabridin (G, 7.5 μg/mL) (**B**), but not to wighteone (W, 3.8 μg/mL) (**A**) and the effect of tryptophan supplementation on rescuing sensitivity in *TAT2* overexpressed *S. cerevisiae* cells exposed to G (**C**). Treated *trp1*Δ, *trp1*Δ’ (=*trp1*Δ + empty vector) strains were cultured the presence of 1 mM tryptophan and/or 7.5 μg/mL glabridin. Values shown are the means ± SD of three biological replicates (each of which is the result of three technical replicates).


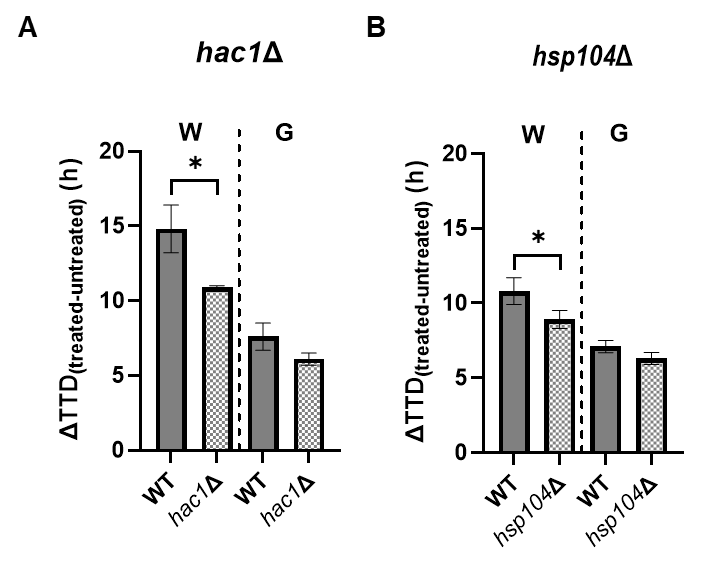


**Figure S8**. Phenotypes of *hac1*Δ and *hsp104*Δ to wighteone (W) and glabridin (G). Treated *S. cerevisiae* *hac1*Δ (**A**) and *hsp104*Δ (**B**) strains were cultured in the presence of 5.0 μg/mL W or 10.0 μg/mL G. Filled bars refer to the WT strain and patterned bars refer to the deletant strains after exposure to prenylated isoflavonoids. Values shown are the means ± SD of three biological replicates (each of which is the result of three technical replicates). Asterisks denote statistically significant differences (p-value < 0.05). For full growth curves, refer to **Figure S9**.

**
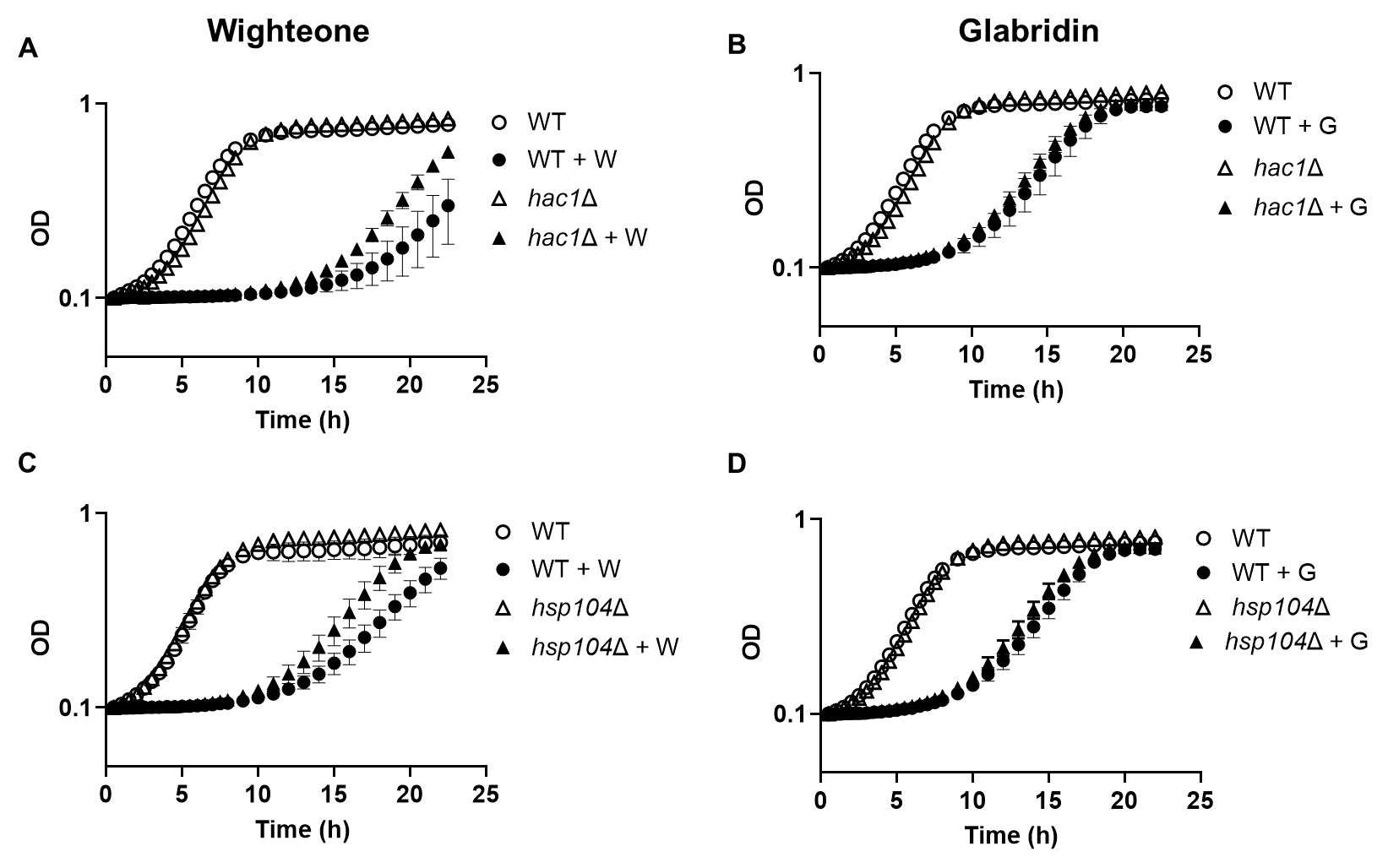
Figure S9**. Full growth curves showing the resistance of *hac1*Δ and *hsp104*Δ to wighteone. Treated S*. cerevisiae* WT, *hac1*Δ (**A**, **B**) and *hsp104*Δ (**C**, **D**) strains were cultured in the presence of 5.0 μg/mL wighteone (**A**, **C**) and 10.0 μg/mL glabridin (**B**, **D**). Values shown are the means ± SD of three biological replicates (each of which is the result of three technical replicates).
